# Author Correction: The ubiquitin ligase RNF5 determines acute myeloid leukemia growth and susceptibility to histone deacetylase inhibitors

**DOI:** 10.1038/s41467-026-73691-z

**Published:** 2026-06-11

**Authors:** Ali Khateb, Anagha Deshpande, Yongmei Feng, Darren Finlay, Joo Sang Lee, Ikrame Lazar, Bertrand Fabre, Yan Li, Yu Fujita, Tongwu Zhang, Jun Yin, Ian Pass, Ido Livneh, Irmela Jeremias, Carol Burian, James R. Mason, Ronit Almog, Nurit Horesh, Yishai Ofran, Kevin Brown, Kristiina Vuori, Michael Jackson, Eytan Ruppin, Aniruddha J. Deshpande, Ze’ev A. Ronai

**Affiliations:** 1 Technion Integrated Cancer Center, Faculty of Medicine, Technion Israel Institute of Technology, Haifa, Israel; 2 Cancer Center, Sanford Burnham Prebys Medical Discovery Institute, La Jolla, CA USA; 3 Cancer Data Science Lab (CDSL), National Cancer Institute, National Institute of Health, Bethesda, MD USA; 4 Laboratory of Translational Genomics, Division of Cancer Epidemiology and Genetics, National Cancer Institute, Bethesda, MD USA; 5 Research Unit Apoptosis in Hematopoietic Stem Cells, Helmholtz Center Munich, German Center for Environmental Health, Munich, Germany; 6 Scripps MD Anderson Cancer Center, La Jolla, CA USA; 7 Rambam Health Care Campus, Epidemiology Department and Biobank, Haifa, Israel; 8 Rambam Health Care Campus, Hematology and Bone marrow Transplantation Department, Haifa, Israel; 9 Present Address: Division of Respiratory Medicine, Department of Internal Medicine, Jikei University School of Medicine, Tokyo, Japan

**Keywords:** Acute myeloid leukaemia, Mechanisms of disease, Ubiquitylation

Correction to: *Nature Communications* 10.1038/s41467-021-25664-7, published online 13 September 2021

The following errors were found in this manuscript and were corrected as follows: 1. Figure 2d was found to have a duplicated image (panels for pLKO and sh#1 were inadvertently duplicated for the MOLM-13 and U937 cell lines). This has been corrected by replacing the MOLM-13 image with the correct data in the updated Figure 2d file. 2. Tabs for Fig. 2 and Fig. 3 in the “Raw Data” Excel file were mislabeled (mistakenly prefixed with 1 (e.g., 1a, 1b, 1c) where they should have been prefixed with “2” (e.g., 2a, 2b, 2c) for Fig. 2 and “3” for Fig. 3. This labeling error has been corrected in the raw data file tabs corresponding to Figures 2 and 3. 3. The excel file for RAW data (tab Fig.2, Fig. 2d), had mistakenly listed the first entry for shRNF5#1 (MOLM-13) as 17.67. This value represents the average of three technical replicates that were rounded now to the integer 18 for clarity and consistency. 4. Molecular weight for RNF5 was mislabeled in Figure 5h and Supplementary Fig. 2h. The correct molecular weight (~15 kDa) has been corrected now in both figures. Molecular weight for RBBP4 was also mislabeled in Figure 5h and has been corrected (~55 kDa). 5. Raw data error for Supplementary Fig. 7c (unintentional duplication for sh#1 resulted in same numbers for row 6 columns 1-3 and row 7 columns 1-3 for the shRNF5#1). This has been now corrected, and the correct values were updated in the revised RAW data file. Correspondingly, Supplementary Fig. 7c has been updated accordingly.

