# Supplementary material for: The ubiquitin ligase RNF5 determines acute myeloid leukemia growth and susceptibility to histone deacetylase inhibitors
Source: Nat Commun. 2021 Sep 13;12:5397. doi: 10.1038/s41467-021-25664-7 (PMC8437979; doi:10.1038/s41467-021-25664-7)
Supplement: Supplementary file 1 — Supplementary Information [file 41467_2021_25664_MOESM1_ESM.pdf]

Supplementary information

The Ubiquitin Ligase RNF5 Determines Acute Myeloid Leukemia Growth and Susceptibility to Histone Deacetylase Inhibitors

Khateb et al.,

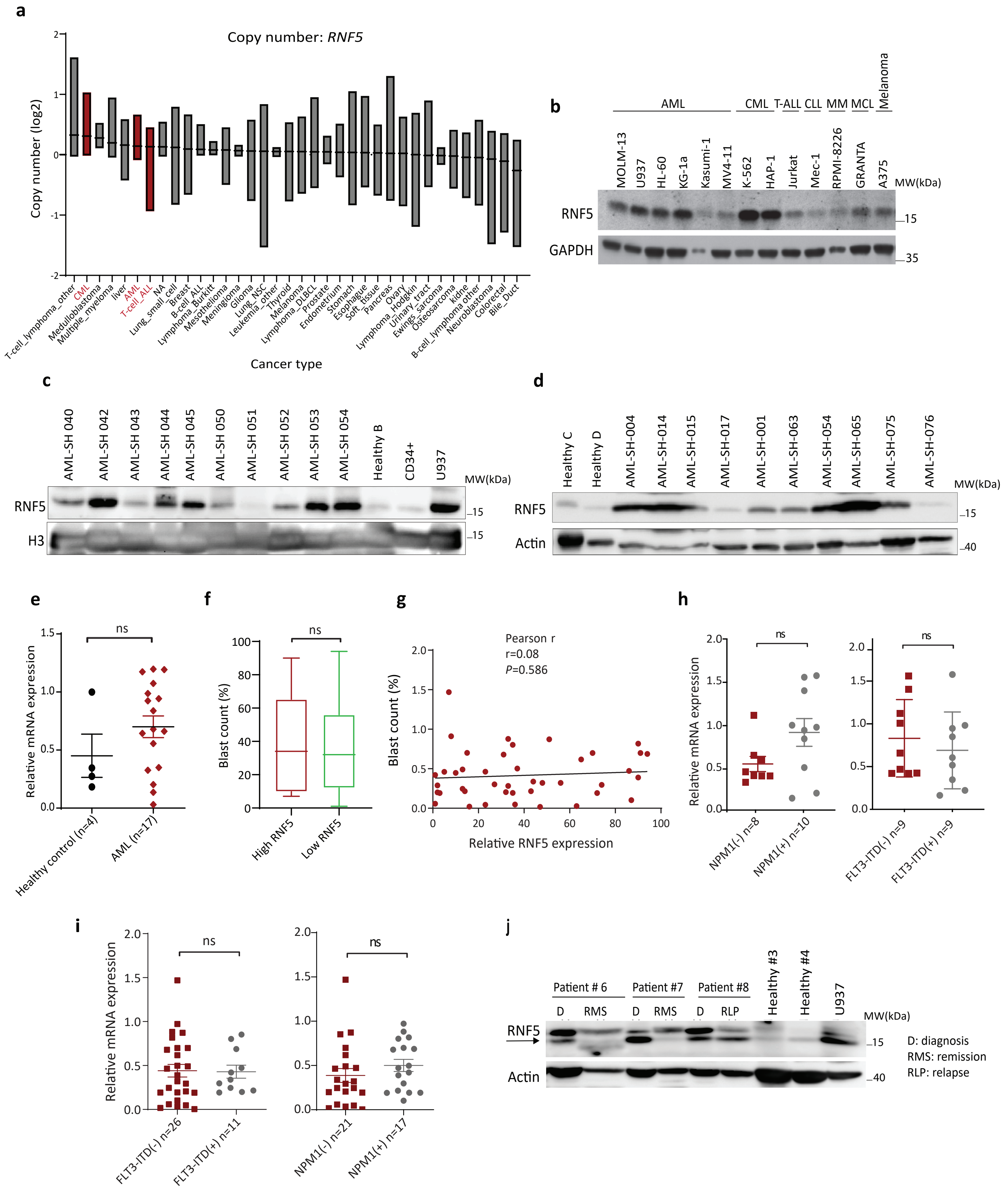
a
Copy number: RNF5
Copy number (log2)
Cancer type
T-cell_lymphoma_other
CML
Medulloblastoma
Multiple_myeloma
liver
AML
T-cell_ALL
NA
Lung_small_cell
Breast
B-cell_ALL
Lymphoma_Burkitt
Mesothelioma
Meningioma
Glioma
Lung_NSC
Leukemia_other
Thyroid
Melanoma
Lymphoma_DLBCL
Prostate
Endometrium
Stomach
Esophague
Soft_tissue
Pancreas
Ovary
Lymphoma_Hodgkin
Urinary_tract
Ewings_sarcoma
Osteosarcoma
kidne
B-cell_lymphoma_other
Neuroblastoma
Colorectal
Bile_Duct
b
AML
CML
T-ALL
CLL
MM
MCL
Melanoma
MOLM-13
U937
HL-60
KG-1a
Kasumi-1
MV4-11
K-562
HAP-1
Jurkat
Mec-1
RPMI-8226
GRANTA
A375
MW(kDa)
RNF5
GAPDH
15
35
c
AML-SH 040
AML-SH 042
AML-SH 043
AML-SH 044
AML-SH 045
AML-SH 050
AML-SH 051
AML-SH 052
AML-SH 053
AML-SH 054
Healthy B
CD34+
U937
MW(kDa)
RNF5
H3
15
15
d
Healthy C
Healthy D
AML-SH-004
AML-SH-014
AML-SH-015
AML-SH-017
AML-SH-001
AML-SH-063
AML-SH-054
AML-SH-065
AML-SH-075
AML-SH-076
MW(kDa)
RNF5
Actin
15
40
e
Relative mRNA expression
ns
Healthy control (n=4)
AML (n=17)
f
Blast count (%)
ns
High RNF5
Low RNF5
g
Pearson r
r=0.08
P=0.586
Blast count (%)
Relative RNF5 expression
h
Relative mRNA expression
ns
NPM1(-) n=8
NPM1(+) n=10
FLT3-ITD(-) n=9
FLT3-ITD(+) n=9
i
Relative mRNA expression
ns
FLT3-ITD(-) n=26
FLT3-ITD(+) n=11
NPM1(-) n=21
NPM1(+) n=17
j
Patient # 6
Patient #7
Patient #8
D
RMS
RLP
Healthy #3
Healthy #4
U937
MW(kDa)
RNF5
Actin
15
40
D: diagnosis
RMS: remission
RLP: relapse

Supplementary Fig. 1: RNF5 protein and transcript levels inversely correlate with AML patient outcome

(a) CCLE data copy number analysis of the *RNF5* locus across cancer cell lines from various tissue sources [1] . Line within the box blot show the mean log2 copy number for each tissue. **(b)** Western blot (WB) analysis of RNF5 in lysates made from indicated cancer cell lines: AML, acute myeloid leukemia; CML, chronic myeloid leukemia; ALL, acute lymphoblastic leukemia; CLL, chronic lymphoblastic leukemia; MM, multiple myeloma; MCL, mantle cell lymphoma; and melanoma. **(c, d)** Abundance of RNF5 and histone H3 in PBMCs and $CD34^+$ from healthy control (Healthy B and CD34+) subjects and AML patients from the Scripps Health Center. **(e)** RT-qPCR analysis of *RNF5* mRNA in PBMCs from healthy control subjects (n=4) and AML patients (n=17) from the Scripps Health Center. Data are presented as the mean ± SEM. P=0.829 by two-tailed unpaired *t*-test. **(f)** Blast count percentages in AML samples expressing high (n=8) versus low (n=35) RNF5 protein. The horizontal band inside boxes indicates the median, the bottom and top edges of the box 25th–75th percentiles and the whiskers indicate the min to max. P=0.254 by two-tailed unpaired *t*-test **(g)** Pearson correlation analysis between percentage of blasts and RNF5 protein levels in AML samples (n=43) from Scripps Health. P=0.586 by two-tailed Pearson Coefficient. **(h)** Relative RNF5 protein levels in AML samples (Scripps Health Center) positive or negative for NPM1. Data are presented as the mean ± SEM. P=0.082 (left) P=0.515 (right) by two-tailed unpaired *t*-test. **(i)** Relative RNF5 protein levels in AML samples (Rambam Health) positive or negative for NPM1 or FLT3 mutations. Data are presented as the mean ± SEM. P=0.926 (left) P=0.296 (right) by two-tailed unpaired *t*-test. **(j)** WB analysis of RNF5 protein in PBMCs from healthy donors or AML patients in the Rambam Center cohort. Arrow indicate RNF5 position. The upper band is unspecific.

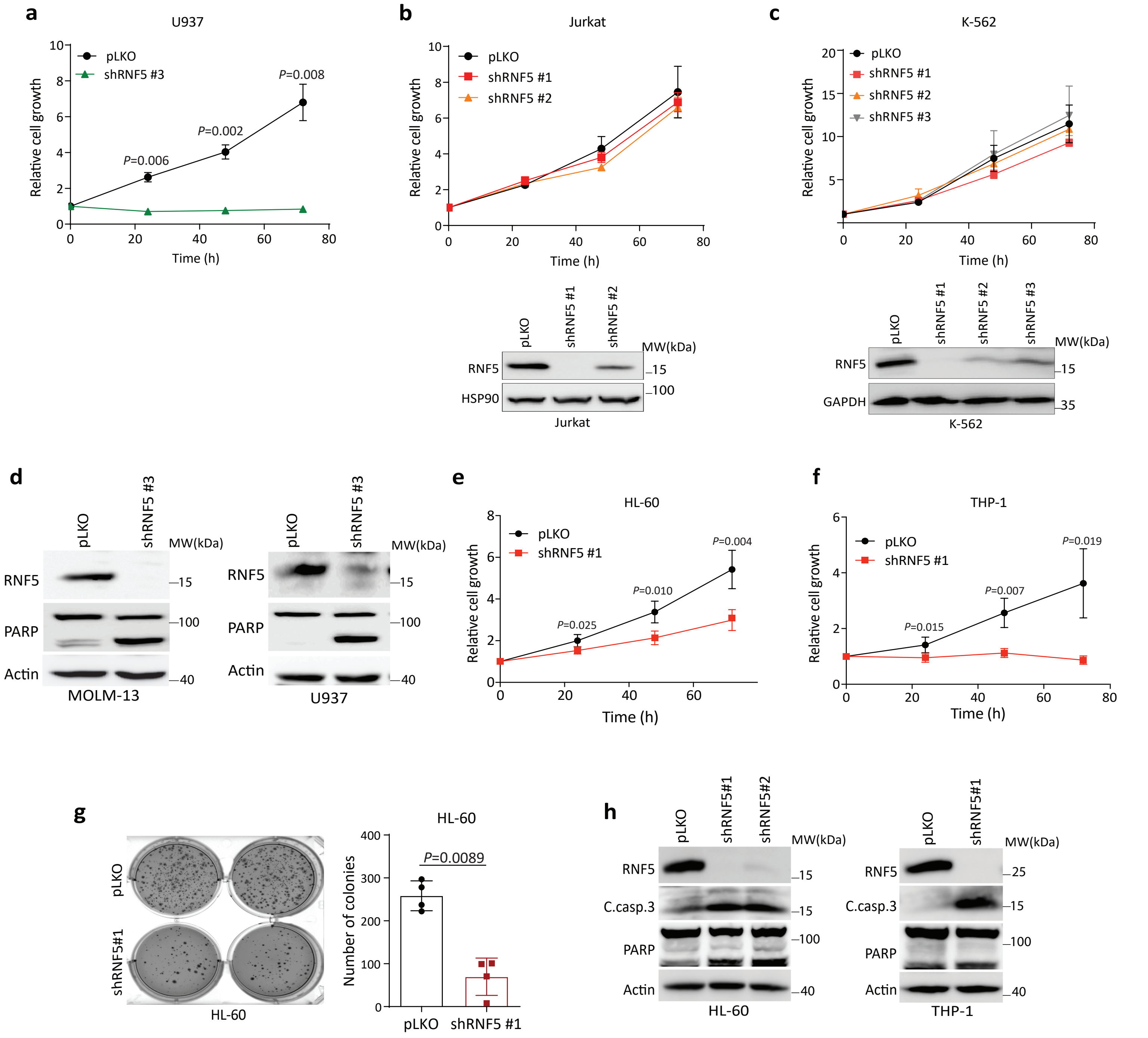

Supplementary Fig. 2: RNF5 is required for AML cell growth

(a) Luminescence-based growth assay of U937 cells expressing empty vector (pLKO) or shRNF5 #3. **(b)** Growth assay of Jurkat cells expressing pLKO or two different shRNF5 constructs. **(c)** Growth assay of K562 cells expressing pLKO or three different shRNF5 constructs. Western blots below B and C show knockdown efficiency. **(d)** Western blot analysis of MOLM-13 and U937 cells 5 days after transduction with pLKO or shRNF5 #3. Data are representative of three experiments. **(e, f)** Luminescence-based growth assays of HL-60 or THP-1 cells transduced with pLKO or shRNF5 #1. **(g)** Plate images (left) and quantification (right) of HL-60 colonies in soft agar. Colonies were assessed after 14 days in culture. **(h)** Western blot analysis of the indicated proteins in HL-60 and THP-1 cells 5 days after transduction with pLKO, shRNF5 #1, or shRNF5 #2. Data are representative of three experiments. Quantified data are presented as the mean ± SD and are representative of n=3 (**a-c**) or n=4 (**e-g**) independent experiments. *P* values were determined using two-tailed paired *t*-test.

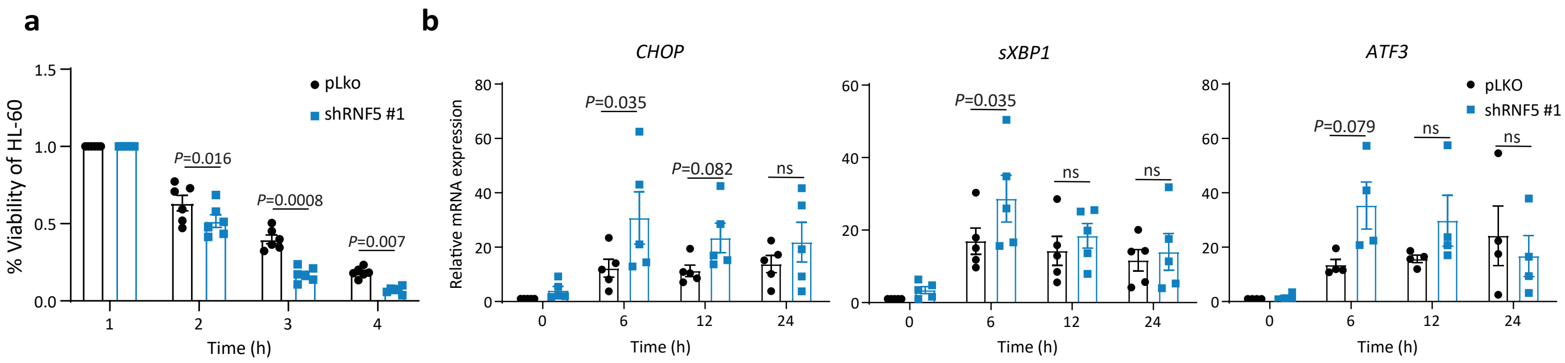

Supplementary Fig. 3: RNF5 KD sensitizes AML cells to ER stress-induced apoptosis

(**a**) Luminescence growth assay of HL-60 cells expressing pLKO or shRNF5 after treatment with tunicamycin (2 μg/mL) for indicated times. Data are presented as mean ± SEM of n=6 independent experiments. **(b)** RT-qPCR analysis of UPR-related genes in HL-60 cells treated with thapsigargin (1 μM) for indicated times. Data are presented as mean ± SEM of n=5 (*CHOP* and *sXBP1*) or n=4 (*ATF3*) independent experiments. *P* values were determined using two-tailed paired *t*-test. ns: not significant.

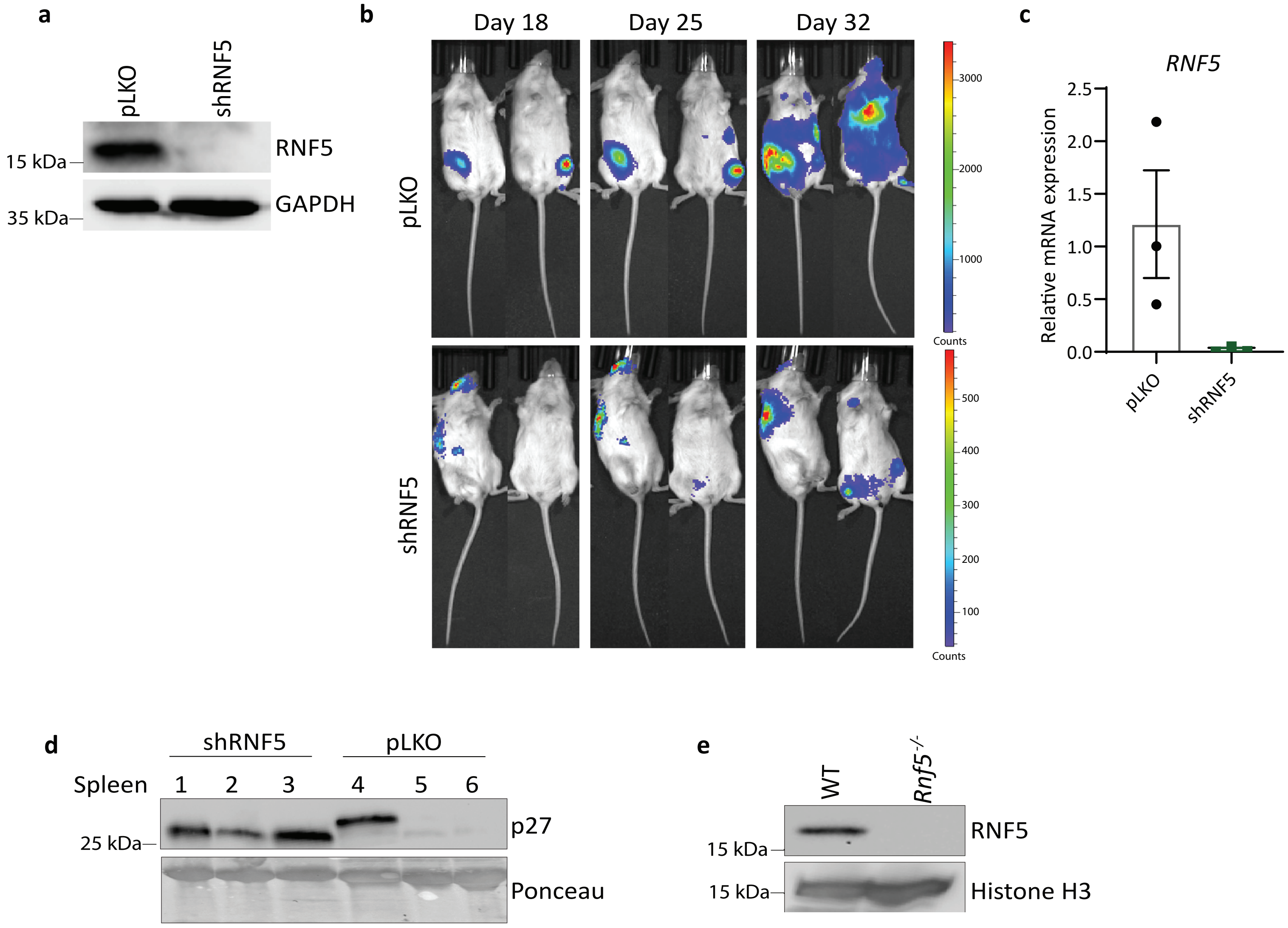

Supplementary Fig. 4: RNF5 suppression antagonizes leukemia establishment and progression *in vivo*

(a) U937-pGFL cells expressing pLKO or inducible shRNF5 were treated 3 days with Dox (1 µg/mL) and then subjected to Western analysis to detect RNF5. GADPH served as a loading control. **(b)** Bioluminescent images of representative mice injected with U937-pGFL expressing empty vector (pLKO) or inducible shRNF5 at days 18, 25 and 32. **(c)** RT-qPCR validation of RNF5 KD from splenocytes of mice injected with pLKO (n=3) or shRNF5 (n=3) cells. Data are presented as mean ± SEM. **(d)** Western blot analysis of p27 in lysates of splenocytes from mice injected with empty vector (pLKO) or shRNF5 cells. Ponceau staining served as loading control. **(e)** Western blot analysis of RNF5 in lysates from WT and *Rnf5*$^{-/-}$ MLL-AF9 transformed cells. H3 served as loading control.

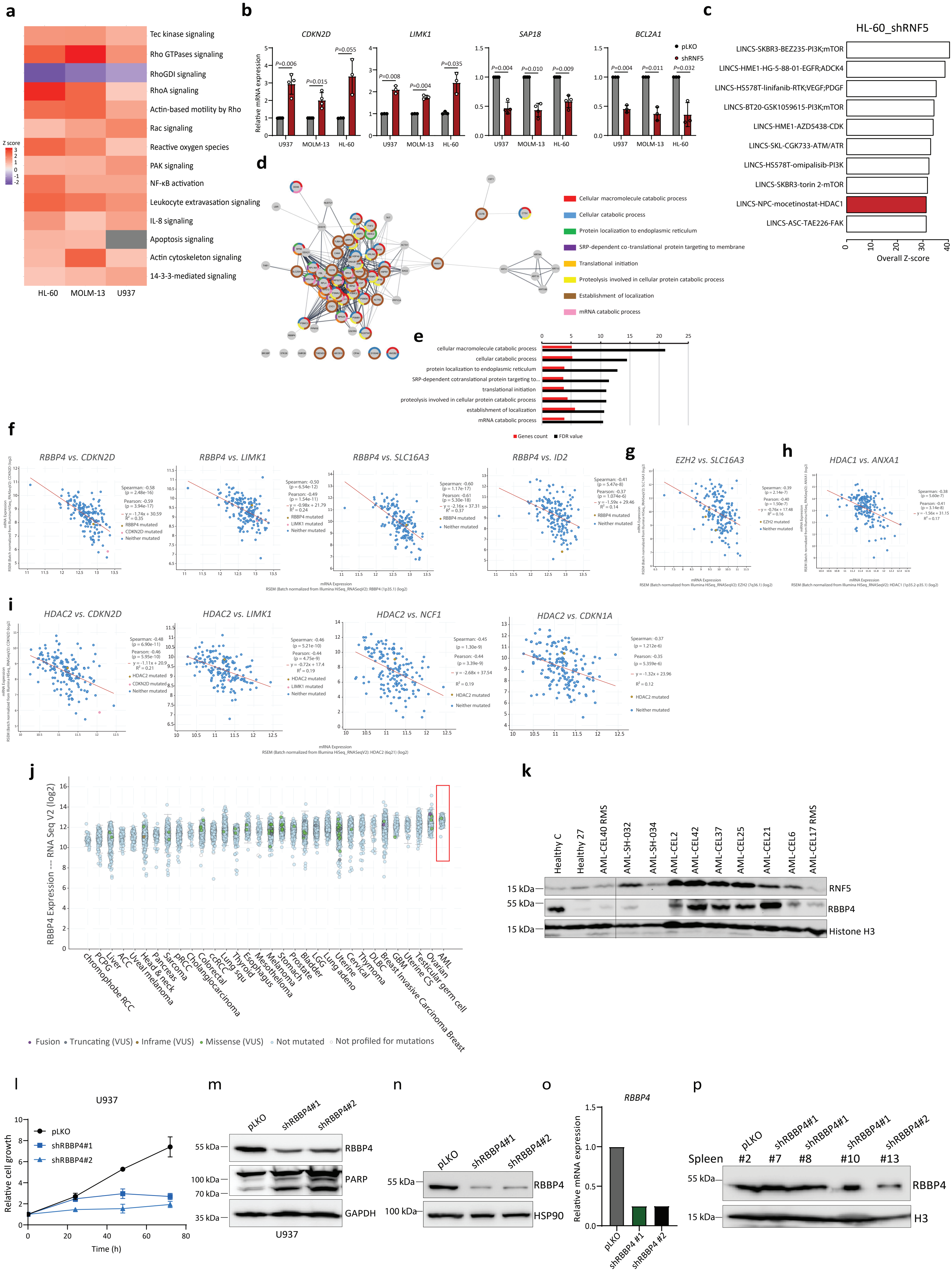
a
Tec kinase signaling
Rho GTPases signaling
RhoGDI signaling
RhoA signaling
Actin-based motility by Rho
Rac signaling
Reactive oxygen species
PAK signaling
NF-κB activation
Leukocyte extravasation signaling
IL-8 signaling
Apoptosis signaling
Actin cytoskeleton signaling
14-3-3-mediated signaling
HL-60
MOLM-13
U937
Z score
b
CDKN2D
LIMK1
SAP18
BCL2A1
Relative mRNA expression
P=0.006
P=0.015
P=0.055
P=0.008
P=0.004
P=0.035
P=0.004
P=0.010
P=0.009
P=0.004
P=0.011
P=0.032
pLKO
shRNF5
U937
MOLM-13
HL-60
c
HL-60_shRNF5
LINCS-SKBR3-BEZ235-PI3K;mTOR
LINCS-HME1-HG-5-88-01-EGFR;ADCK4
LINCS-HS578T-linifanib-RTK;VEGF;PDGF
LINCS-BT20-GSK1059615-PI3K;mTOR
LINCS-HME1-AZD5438-CDK
LINCS-SKL-CGK733-ATM/ATR
LINCS-HS578T-omipalisib-PI3K
LINCS-SKBR3-torin 2-mTOR
LINCS-NPC-mocetinostat-HDAC1
LINCS-ASC-TAE226-FAK
Overall Z-score
d
Cellular macromolecule catabolic process
Cellular catabolic process
Protein localization to endoplasmic reticulum
SRP-dependent co-translational protein targeting to membrane
Translational initiation
Proteolysis involved in cellular protein catabolic process
Establishment of localization
mRNA catabolic process
e
cellular macromolecule catabolic process
cellular catabolic process
protein localization to endoplasmic reticulum
SRP-dependent cotranslational protein targeting to...
translational initiation
proteolysis involved in cellular protein catabolic process
establishment of localization
mRNA catabolic process
Genes count
FDR value
f
RBBP4 vs. CDKN2D
RBBP4 vs. LIMK1
RBBP4 vs. SLC16A3
RBBP4 vs. ID2
mRNA Expression
RSEM (Batch normalized from Illumina HiSeq_RNASeqV2): RBBP4 (1p35.1) (log2)
g
EZH2 vs. SLC16A3
mRNA Expression
RSEM (Batch normalized from Illumina HiSeq_RNASeqV2): EZH2 (7q36.1) (log2)
h
HDAC1 vs. ANXA1
mRNA Expression
RSEM (Batch normalized from Illumina HiSeq_RNASeqV2): HDAC1 (1p35.2-p35.1) (log2)
i
HDAC2 vs. CDKN2D
HDAC2 vs. LIMK1
HDAC2 vs. NCF1
HDAC2 vs. CDKN1A
mRNA Expression
RSEM (Batch normalized from Illumina HiSeq_RNASeqV2): HDAC2 (6q21) (log2)
j
RBBP4 Expression --- RNA Seq V2 (log2)
chromophobe RCC
PCPG
Liver
ACC
Uveal melanoma
Head & neck
Pancreas
Sarcoma
pRCC
Cholangiocarcinoma
Colorectal
ccRCC
Lung squ
Thyroid
Esophagus
Mesothelioma
Melanoma
Stomach
Prostate
Bladder
LGG
Lung adeno
Uterine
Cervical
Thymoma
DLBC
Breast Invasive Carcinoma Breast
GBM
Uterine CS
Testicular germ cell
Ovarian
AML
Fusion
Truncating (VUS)
Inframe (VUS)
Missense (VUS)
Not mutated
Not profiled for mutations
k
Healthy C
Healthy 27
AML-CEL40 RMS
AML-SH-032
AML-SH-034
AML-CEL2
AML-CEL42
AML-CEL37
AML-CEL25
AML-CEL21
AML-CEL6
AML-CEL17 RMS
15 kDa
55 kDa
15 kDa
RNF5
RBBP4
Histone H3
l
U937
pLKO
shRBBP4#1
shRBBP4#2
Relative cell growth
Time (h)
m
pLKO
shRBBP4#1
shRBBP4#2
55 kDa
100 kDa
70 kDa
35 kDa
RBBP4
PARP
GAPDH
U937
n
pLKO
shRBBP4#1
shRBBP4#2
55 kDa
100 kDa
RBBP4
HSP90
o
RBBP4
Relative mRNA expression
pLKO
shRBBP4 #1
shRBBP4 #2
p
pLKO
shRBBP4#1
shRBBP4#1
shRBBP4#1
shRBBP4#2
Spleen
#2
#7
#8
#10
#13
55 kDa
15 kDa
RBBP4
H3

Supplementary Fig. 5: RNF5 activity modulates transcription in AML cells

(a) Top canonical pathways identified by Ingenuity Pathway Analysis comparing genes differentially expressed in indicated AML cell lines upon RNF5-KD. **(b)** RT-qPCR analysis of a select subset of genes identified as deregulated upon RNF5-KD by RNA-seq analysis. Data are presented as the mean ± SD of n=4 (*CDKN2D*) or n=3 (*LIMK1*, *SAP18*, and *BCL2A1*) independent experiments. **(c)** Top ten drug screening results from LINCS database matched with transcriptomic changes in shRNF5 HL-60 line. Values are overall z-scores from IPA Analysis Match database. HDAC1 inhibitor results are shown in red. **(d)** RNF5 interaction network generated from immunoprecipitation data and Cytoscape. Colors correspond to indicated pathways. **(e)** Pathway enrichment analysis displaying gene counts (log2 transformed) and the corresponding false discovery rate (-log10 transformed) for each pathway**. (f-i)** Co-expression of *RBBP4* (f), *EZH2* (g), *HDAC1* (h) or *HDAC2* (i) mRNA and the indicated RNF5 target genes in AML analyzed in cBioPortal using data from TCGA. Pearson correlation, $P<0.0001$, n=165. **(j)** Analysis of RBBP4 expression in different human cancers from the cBioPortal using data from TCGA. **(k)** Western blot analysis of RBBP4 in PBMCs from healthy control subjects and AML patients from Scripps Health and Rambam Medical Center cohorts. RMS, remission. **(l)** Growth assay of U937 cells after transduction with empty vector (pLKO) or the indicated shRBBP4 constructs. Data are presented as the mean ± SD of two independent experiments. **(m)** Western blot analysis of indicated proteins in U937 cells expressing empty vector (pLKO) or two different shRBBP4 constructs. **(n)** Western blot confirmation of RBBP4 KD in U937-pGFL cells used for the xenograft experiment. **(o)** RT-qPCR confirmation of RBBP4 KD in U937-pGFL cells used for the xenograft experiment. **(p)** Western blot analysis of RBBP4 in lysates of splenocytes from mice injected with empty vector (pLKO) or shRBBP4 cells. *P* values were determined using two-tailed paired *t*-test.

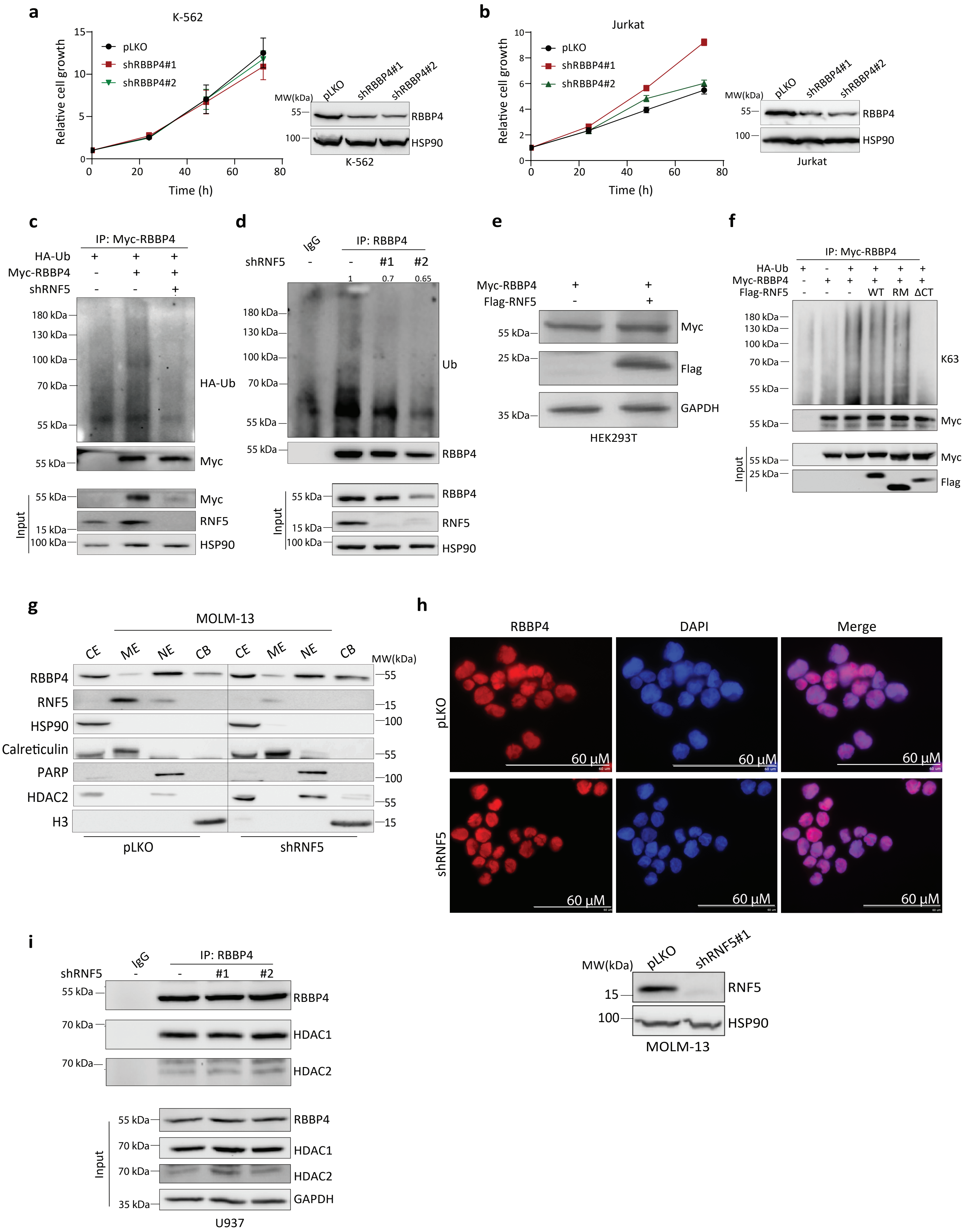
a
K-562
Relative cell growth
Time (h)
pLKO
shRBBP4#1
shRBBP4#2
MW(kDa)
RBBP4
HSP90
K-562
b
Jurkat
Relative cell growth
Time (h)
pLKO
shRBBP4#1
shRBBP4#2
MW(kDa)
RBBP4
HSP90
Jurkat
c
IP: Myc-RBBP4
HA-Ub
Myc-RBBP4
shRNF5
HA-Ub
Myc
Input
Myc
RNF5
HSP90
d
IgG
IP: RBBP4
shRNF5
#1
#2
1
0.7
0.65
Ub
RBBP4
Input
RBBP4
RNF5
HSP90
e
Myc-RBBP4
Flag-RNF5
Myc
Flag
GAPDH
HEK293T
f
IP: Myc-RBBP4
HA-Ub
Myc-RBBP4
Flag-RNF5
WT
RM
ΔCT
K63
Myc
Input
Myc
Flag
g
MOLM-13
CE
ME
NE
CB
MW(kDa)
RBBP4
RNF5
HSP90
Calreticulin
PARP
HDAC2
H3
pLKO
shRNF5
h
RBBP4
DAPI
Merge
pLKO
shRNF5
60 μM
i
IgG
IP: RBBP4
shRNF5
#1
#2
RBBP4
HDAC1
HDAC2
Input
RBBP4
HDAC1
HDAC2
GAPDH
U937
MW(kDa)
pLKO
shRNF5#1
RNF5
HSP90
MOLM-13

Supplementary Fig. 6: RNF5 interacts with and ubiquitinates RBBP4

(a) Growth assay of K-562 cells following transduction with empty vector (pLKO) or the indicated shRBBP4 constructs. Western blot shows knockdown efficiency. Data are presented as the mean ± SD of n=3 independent experiment. **(b)** Growth assay of Jurkat cells after transduction with pLKO or the indicated shRBBP4 constructs. Western blot shows knockdown efficiency. Data are presented as the mean ± SD of n=2 independent experiment. **(c)** Western blot analysis of anti-Myc immunoprecipitates and lysates of HEK293T cells co-expressing Myc-RBBP4, HA-Ub, and shRNF5. Cells were treated with MG132 (10 μm) for 4 h before lysis. **(d)** Western blot analysis of anti-RBBP4 immunoprecipitates and lysates of MOLM-13 cells expressing the indicated shRNF5 constructs. Cells were treated with MG132 (10 μm) for 4 h before lysis. Quantification of the ubiquitination smear relative to the amount of RBBP4 pull down is shown at the top. **(e)** Western blot analysis of anti-Myc immunoprecipitates and lysates of HEK293T cells co-expressing Myc-RBBP4, HA-Ub, and the indicated Flag-tagged RNF5 constructs. Cells were treated with MG132 (10 μm) 4 h before lysis. **(f)** Western blot analysis of indicated proteins in HEK293T cells transfected with Myc-RBBP4 and Flag-RNF5. **(g)** Western blot analysis of RBBP4 and RNF5 in indicated fractions of MOLM-13 cells expressing pLKO or shRNF5 #1. CE, cytoplasmic extract; ME, membrane extract; NE, nuclear extract; CB, chromatin bound. Histone H3, HSP90, and calreticulin serve as controls for chromatin, cytosol, and membrane fractions, respectively. **(h)** Immunofluorescence staining of RBBP4 (red) in control or shRNF5-expressing MOLM-13 cells. Nuclei were stained with DAPI (blue). Scale bar 60μM. Western blot below shows RNF5-KD efficiency. **(i)** Immunoprecipitation and Western blot analysis of the interaction of RBBP4 with HDAC1, HDAC2, or EZH2 in U937 cells expressing indicated constructs. Cells were treated with MG132 (10 μm) 4 h before lysis.

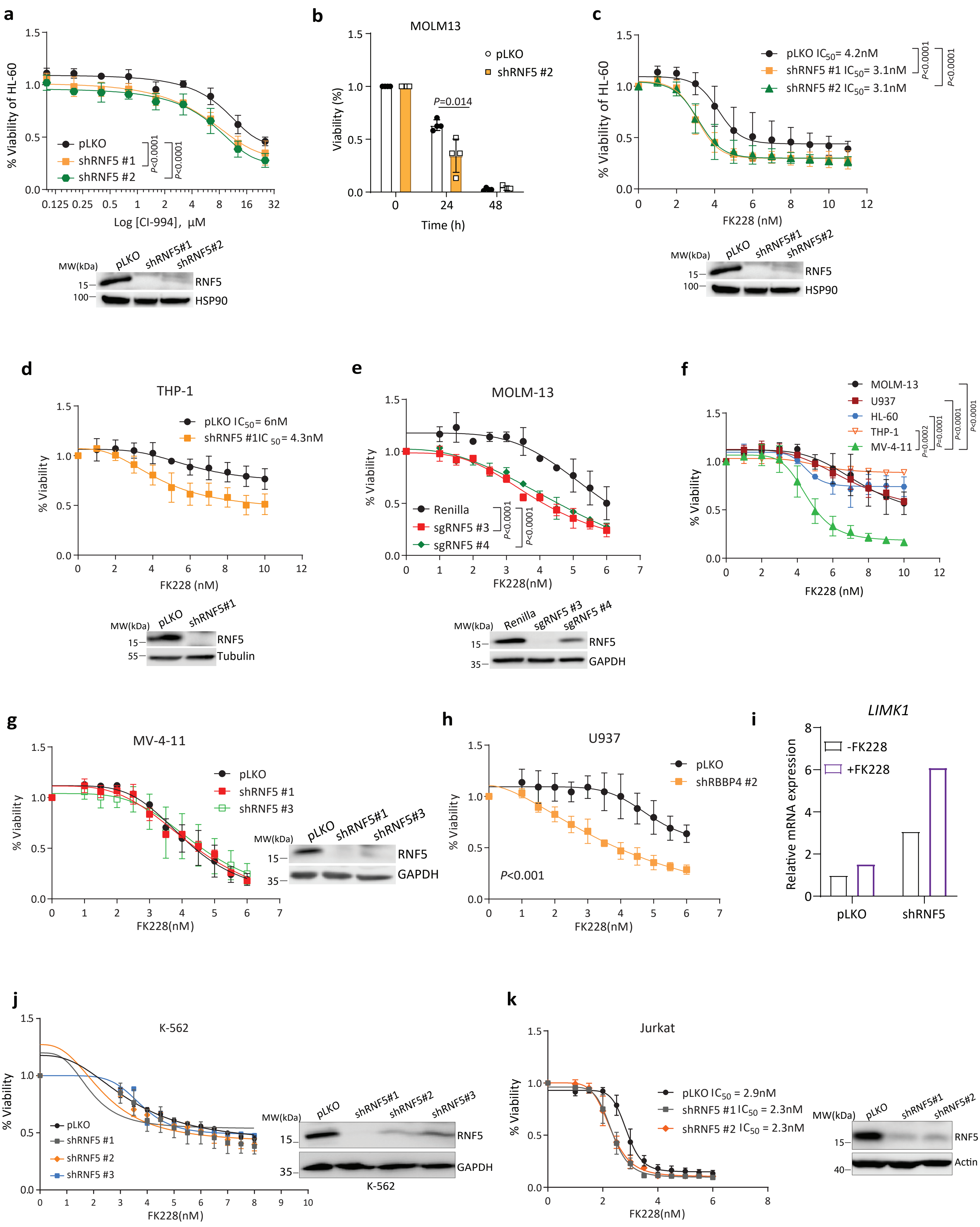
a
% Viability of HL-60
pLKO
shRNF5 #1
shRNF5 #2
P<0.0001
P<0.0001
Log [CI-994], µM
MW(kDa)
pLKO
shRNF5#1
shRNF5#2
RNF5
HSP90
b
MOLM13
pLKO
shRNF5 #2
Viability (%)
P=0.014
Time (h)
c
% Viability of HL-60
pLKO IC50= 4.2nM
shRNF5 #1 IC50= 3.1nM
shRNF5 #2 IC50= 3.1nM
FK228 (nM)
d
THP-1
pLKO IC50= 6nM
shRNF5 #1IC 50= 4.3nM
% Viability
FK228 (nM)
Tubulin
e
MOLM-13
Renilla
sgRNF5 #3
sgRNF5 #4
FK228(nM)
GAPDH
f
MOLM-13
U937
HL-60
THP-1
MV-4-11
P=0.0002
P=0.0001
g
MV-4-11
shRNF5 #3
h
U937
shRBBP4 #2
P<0.001
i
LIMK1
-FK228
+FK228
Relative mRNA expression
shRNF5
j
K-562
k
Jurkat
pLKO IC50 = 2.9nM
shRNF5 #1 IC50 = 2.3nM
shRNF5 #2 IC50 = 2.3nM
Actin

Supplementary Fig. 7: RNF5 KD sensitizes AML cells to HDAC inhibition

(a) Viability of HL-60 cells expressing pLKO or two shRNF5 constructs after treatment for 24 h with CI-994. Western blot below confirms RNF5-KD. **(b)** Viability of MOLM-13 cells after treatment for 24 h with 3.5 nM FK228. **(c, d)** Viability of HL-60 (c) or THP-1(d) cells expressing pLKO or shRNF5 constructs after treatment for 24 h with FK228. Western blot below confirms RNF5-KD. **(e)** Viability of MOLM-13 cells stably expressing Cas9 and transduced with control *Renilla*-or RNF5-targeting sgRNA and treated for 24 h with FK228. Western blot shows reduction in RNF5 levels. **(f)** Viability of MOLM-13, U937, MV-4-11, THP-1, and HL-60 cells after treatment for 24 h with FK228. **(g)** Viability of MV-4-11 cells expressing pLKO or shRNF5 constructs and treated for 24 h with indicated FK228 concentrations. Data are presented as the mean ± SD of n=2 independent experiments. **(h)** Viability of U937 cells expressing pLKO or shRBBP4 and treated for 24 h with the indicated FK228 concentrations. **(i)** RT-qPCR analysis of *LIMK1* mRNA in MOLM-13 cells expressing empty vector (pLKO) or shRNF5 #1 and treated 15 h with 4nM FK228. Data are presented as the mean ± SD of n=2 independent experiments. **(j)** Viability of K-562 cells expressing pLKO or three shRNF5 constructs after treatment for 30 h with the indicated FK228 concentrations. Western blot confirms RNF5-KD. (**k)** Viability of Jurkat cells expressing pLKO or two shRNF5 constructs after treatment for 24 h with indicated FK228 concentrations. Western blot confirms RNF5-KD. Quantified data are presented as the mean ± SD of n=3 (**a**, **e-h**, and **j**) or n=4 (**b-d**) independent experiments. *P* values were determined using two-tailed *t*-test (**b**) or two-way ANOVA (**h**) followed by Tukey's multiple comparison test (**a**, **c**, **e** and **f**).

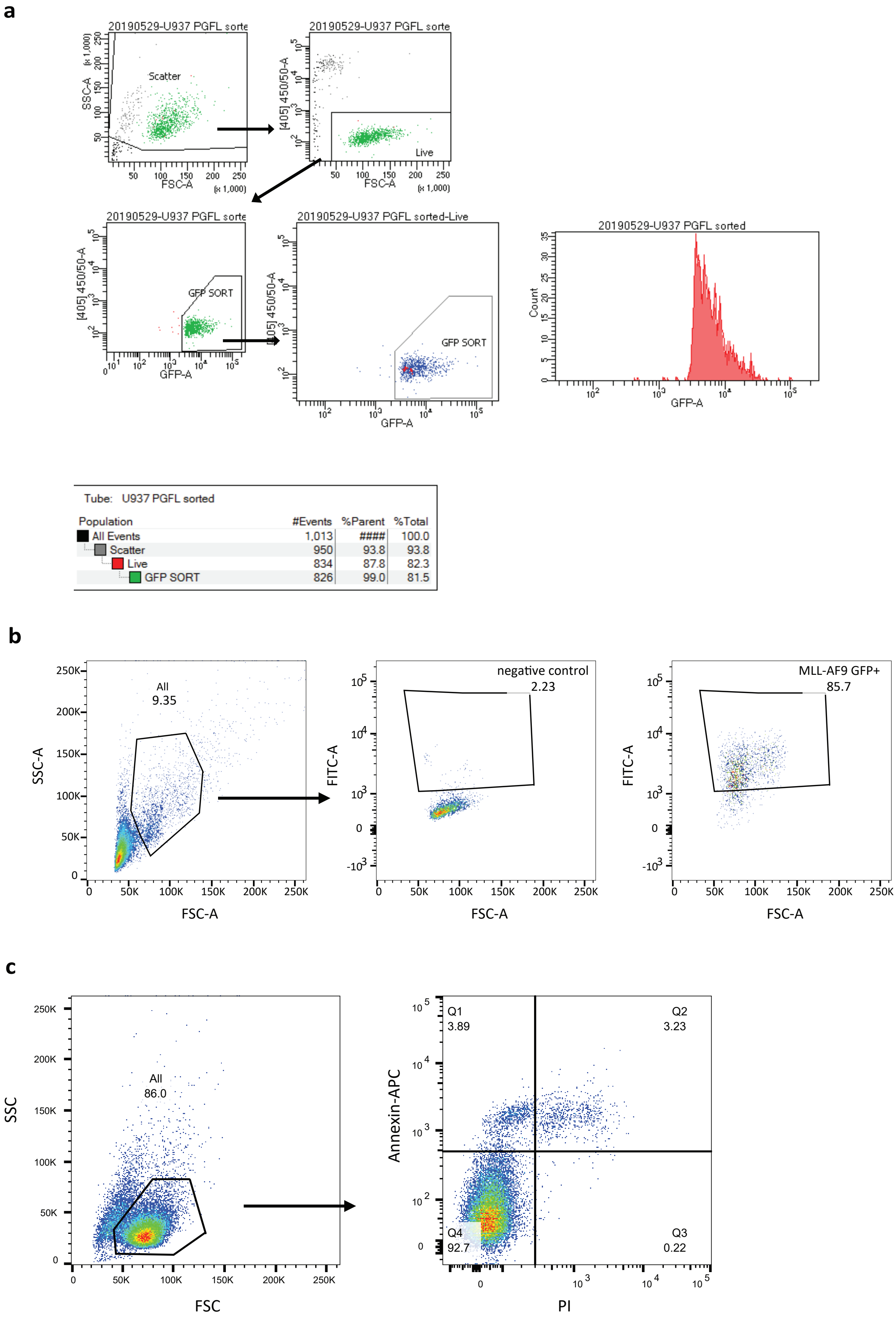

Supplementary Fig. 8: Gating strategies for FACS analysis experiments

(a) A representative example of gating used for sorting of U937-pGFL and transformed MLL-AF9 GFP+ cells. Related to Fig. 4a, c. **(b)** Gating strategy of GFP+ cells quantification in peripheral blood of mice intravenously injected with GFP-MLL-AF9–transformed cells (Fig. 4g). **(c)** Gating strategy for Annexin-V/PI staining (Fig. 3f).

Supplementary References

1 Barretina, J. et al. The Cancer Cell Line Encyclopedia enables predictive modelling of anticancer drug sensitivity. Nature 483, 603-607, doi:10.1038/nature11003 (2012).
