# Supplementary material for: The ubiquitin ligase RNF5 determines acute myeloid leukemia growth and susceptibility to histone deacetylase inhibitors
Source: Nat Commun. 2021 Sep 13;12:5397. doi: 10.1038/s41467-021-25664-7 (PMC8437979; doi:10.1038/s41467-021-25664-7)
Supplement: Supplementary file 3 — Description of Additional Supplementary Files [file 41467_2021_25664_MOESM3_ESM.pdf]

Description of Additional Supplementary Files

Supplementary Data 1. Genes commonly deregulated upon RNF5 knockdown in AML cell lines (HL-60, MOLM-13, and U937)

Supplementary Data 2. Top screening results from the LINCS database matched with transcriptomic data from MOLM-13 or HL-60 cell lines following RNF5 KD

Supplementary Data 3. List of RNF5 interacting proteins identified by LC-MS/MS
