# Supplementary material for: The ubiquitin ligase RNF5 determines acute myeloid leukemia growth and susceptibility to histone deacetylase inhibitors
Source: Nat Commun. 2021 Sep 13;12:5397. doi: 10.1038/s41467-021-25664-7 (PMC8437979; doi:10.1038/s41467-021-25664-7)
Supplement: Supplementary file 7 — Reporting Summary [file 41467_2021_25664_MOESM7_ESM.pdf]

nature portfolio

Corresponding author(s): Ze'ev Ronai

Last updated by author(s): Jul 30, 2021

Reporting Summary

Nature Portfolio wishes to improve the reproducibility of the work that we publish. This form provides structure for consistency and transparency in reporting. For further information on Nature Portfolio policies, see our Editorial Policies and the Editorial Policy Checklist.

Statistics

For all statistical analyses, confirm that the following items are present in the figure legend, table legend, main text, or Methods section.

n/a	Confirmed	
☐	☒	The exact sample size (*n*) for each experimental group/condition, given as a discrete number and unit of measurement
☐	☒	A statement on whether measurements were taken from distinct samples or whether the same sample was measured repeatedly
☐	☒	The statistical test(s) used AND whether they are one- or two-sided<br>*Only common tests should be described solely by name; describe more complex techniques in the Methods section.*
☐	☒	A description of all covariates tested
☐	☒	A description of any assumptions or corrections, such as tests of normality and adjustment for multiple comparisons
☐	☒	A full description of the statistical parameters including central tendency (e.g. means) or other basic estimates (e.g. regression coefficient) AND variation (e.g. standard deviation) or associated estimates of uncertainty (e.g. confidence intervals)
☐	☒	For null hypothesis testing, the test statistic (e.g. *F*, *t*, *r*) with confidence intervals, effect sizes, degrees of freedom and *P* value noted<br>*Give P values as exact values whenever suitable.*
☒	☐	For Bayesian analysis, information on the choice of priors and Markov chain Monte Carlo settings
☒	☐	For hierarchical and complex designs, identification of the appropriate level for tests and full reporting of outcomes
☐	☒	Estimates of effect sizes (e.g. Cohen's *d*, Pearson's *r*), indicating how they were calculated

Our web collection on statistics for biologists contains articles on many of the points above.

Software and code

Policy information about availability of computer code

Data collection	RNA-seq bar-coded libraries were constructed using the NEBNext Ultra™ Directional RNA Library Prep Kit for Illumina (NEB, Ipswich, MA). Libraries were pooled and single end-sequenced (1×75) on the Illumina NextSeq 500 using the High output V2 kit (Illumina, San Diego, CA). Flow cytomtrey data: All data were collected on an LSRFortessa (BD Biosciences).
Data analysis	Data were analyzed using GraphPad Prism 8 and 9 (graphing, statistical analysis), FlowJo v10.1 and v9.9 (flow cytometry), FASTQC (v0.11.5), https://www.bioinformatics.babraham.ac.uk/projects/fastqc/; CUTADAPT (v1.1), https://github.com/marcelm/cutadapt; STAR (v2.5.2a): https://github.com/alexdobin/STAR; Subread (v1.50), http://subread.sourceforge.net; SARTools (v1.2.0), https://github.com/PF2-pasteur-fr/SARTools; edgeR (v3.34.0), https://bioconductor.org/packages/release/bioc/html/edgeR.html; RSEM (v1.3.2), https://deweylab.github.io/RSEM/; Ingenuity Pathway Analysis (IPA), http://www.ingenuity.com; MaxQuant (v1.5.5.0); Crapome (version 2.0); Cytoscape (version 3.8.1).

For manuscripts utilizing custom algorithms or software that are central to the research but not yet described in published literature, software must be made available to editors and reviewers. We strongly encourage code deposition in a community repository (e.g. GitHub). See the Nature Portfolio guidelines for submitting code & software for further information.

Data

Policy information about availability of data

All manuscripts must include a data availability statement. This statement should provide the following information, where applicable:

- Accession codes, unique identifiers, or web links for publicly available datasets
- A description of any restrictions on data availability
- For clinical datasets or third party data, please ensure that the statement adheres to our policy

The RAW MS data have been deposited in the MassIVE repository under accession code MSV000083160, https://massive.ucsd.edu/ProteoSAFe/dataset.jsp?

task=321eefef71fe4baa8900da284d5f66f3). RNA-Seq RAW data in FASTQ format from RNF5 knock-down experiments have been deposited in the NCBI Gene Expression Omnibus (GEO) database under access code GSE155929, https://www.ncbi.nlm.nih.gov/geo/query/acc.cgi?acc=GSE155929. All other data are available within the article and its Supplementary Information. The TCGA and LINCS databases used in this study were processed by Qiagen Ingenuity Pathway Analysis. The raw data of TCGA database are available through GDC Data Portal (https://portal.gdc.cancer.gov/). The raw data of LINCS are available through NIH LINCS (https://lincsproject.org/). Source data are provided with this paper.

Field-specific reporting

Please select the one below that is the best fit for your research. If you are not sure, read the appropriate sections before making your selection.

- [x] Life sciences
- [ ] Behavioural & social sciences
- [ ] Ecological, evolutionary & environmental sciences

For a reference copy of the document with all sections, see nature.com/documents/nr-reporting-summary-flat.pdf

Life sciences study design

All studies must disclose on these points even when the disclosure is negative.

Sample size	For animal experiments, using 10 animals per each of our experimental groups, power analysis indicate the probability is 90 percent that each trial will detect a treatment difference at a two-sided 0.05 significance level, if the true difference between treatments is 1.389 times the standard deviation of the measured parameters for each trial. Unexpectedly dead animals were excluded. For in vitro experiments, a minimum of 3 samples were chosen as a sample size to ensure adequate power, unless stated otherwise. Sample sizes and statistical data are reported in figure legends.
Data exclusions	The data from unexpectedly dead animals were excluded. No data were excluded from in vitro analysis.
Replication	Experiments were repeated at least 2 or 3 times independently (unless otherwise stated) and were reproducible. The number of repeats were indicated in figure legends.
Randomization	For in vitro studies, randomization is not applicable as cells with different treatments or genetic knockdown cannot be randomized. For animal experiments, the mice were randomly assigned into different experimental groups whenever possible, except in experiments required specific genotypes.
Blinding	The investigators were blinded to group allocation during experiments and outcome assessment.

Reporting for specific materials, systems and methods

We require information from authors about some types of materials, experimental systems and methods used in many studies. Here, indicate whether each material, system or method listed is relevant to your study. If you are not sure if a list item applies to your research, read the appropriate section before selecting a response.

Materials & experimental systems

n/a	Involved in the study	
☐	☒	Antibodies
☐	☒	Eukaryotic cell lines
☒	☐	Palaeontology and archaeology
☐	☒	Animals and other organisms
☐	☒	Human research participants
☒	☐	Clinical data
☒	☐	Dual use research of concern

Methods

n/a	Involved in the study	
☒	☐	ChIP-seq
☐	☒	Flow cytometry
☒	☐	MRI-based neuroimaging

Antibodies

Antibodies used

Cell Signaling Technology: Cleaved caspase 3 (#9661), p27 (#3688), p21 (#2947), PARP(#9532), Myc-Tag (#2276), HDAC1 (#2062), HDAC2 (#57156), EZH2 (#5246), Actin (#4970), Ubiquitin (#3936), K63-linkage Specific Polyubiquitin (#5621), Acetyl-Histone H3 (Lys9) (#9649),Tri-Methyl-Histone H3 (Lys27) (#9733), Histone H3 (#9717)
Biolegend: HA-Tag (901501), Annexin V-APC (640919)
Abcam: GAPDH (Ab8245), H3K27ac (Ab3594)
Sigma-Aldrich: Flag-Tag (F1804), Tubulin (T9026)
Santa Cruz: RNF5 (sc-81716), HSP90 (sc-13119), Caspase 3 (sc-56053), Calregulin (sc-166837)
Novus Biologicals: RBBP4 (NBP1-41201)
HRP-conjugated secondary antibodies were from Jackson ImmunoResearch (goat-anti-mouse-HRP (AB_2338504) and goat-anti-rabbit-HRP (AB_2337938)
Antibodies are also listed in the Methods section under their respective experimental method.

Validation

The specificity of the antibodies purchased from commercial sources were validated by the manufacturer as noted on their website (links provided below for each antibody). We also validated some antibodies by detecting proteins at the expected sizes, which were consistent with the literature reports. Additionally, our use of shRNAs validated some antibodies (as noted below):

Cleaved caspase 3
Validation Refs. from the manufacturer's website: https://www.cellsignal.com/products/primary-antibodies/cleaved-caspase-3-asp175-antibody/9661

p27
Validation Refs. from the manufacturer's website: https://www.cellsignal.com/products/primary-antibodies/p27-kip1-d37h1-rabbit-mab/3688

p21
Validation Refs. from the manufacturer's website: https://www.cellsignal.com/products/primary-antibodies/p21-waf1-cip1-12d1-rabbit-mab/2947?site-search-type=Products&N=4294956287&Ntt=p21+%28%232947%29&fromPage=plp&_requestid=251126

PARP
Validation Refs. from the manufacturer's website: https://www.cellsignal.com/products/primary-antibodies/parp-46d11-rabbit-mab/9532?site-search-type=Products&N=4294956287&Ntt=parp&fromPage=plp

Myc-Tag
Validation Refs. from the manufacturer's website: https://www.cellsignal.com/products/primary-antibodies/myc-tag-9b11-mouse-mab/2276

HDAC1
Validation Refs. from the manufacturer's website: https://www.cellsignal.com/products/primary-antibodies/histone-deacetylase-1-hdac1-antibody/2062?site-search-type=Products&N=4294956287&Ntt=hdac1+%232062&fromPage=plp&_requestid=251409

HDAC2
Validation Refs. from the manufacturer's website: https://www.cellsignal.com/products/primary-antibodies/hdac2-d6s5p-rabbit-mab/57156?site-search-type=Products&N=4294956287&Ntt=%28%2357156%29&fromPage=plp&_requestid=251431

EZH2
Validation Refs. from the manufacturer's website: https://www.cellsignal.com/products/primary-antibodies/ezh2-d2c9-xp-rabbit-mab/5246?site-search-type=Products&N=4294956287&Ntt=%28%235246%29&fromPage=plp&_requestid=251478

Actin
Validation Refs. from the manufacturer's website: https://www.cellsignal.com/products/primary-antibodies/b-actin-13e5-rabbit-mab/4970

Ubiquitin
Validation Refs. from the manufacturer's website: https://www.cellsignal.com/products/primary-antibodies/ubiquitin-p4d1-mouse-mab/3936?site-search-type=Products&N=4294956287&Ntt=ubiquitin&fromPage=plp

K63-linkage Specific Polyubiquitin
Validation Refs. from the manufacturer's website: https://www.cellsignal.com/products/primary-antibodies/k63-linkage-specific-polyubiquitin-d7a11-rabbit-mab/5621?site-search-type=Products&N=4294956287&Ntt=63-linkage+specific+polyubiquitin+%28%23562&fromPage=plp

Acetyl-Histone H3 (Lys9)
Validation Refs. from the manufacturer's website: https://www.cellsignal.com/products/primary-antibodies/acetyl-histone-h3-lys9-c5b11-rabbit-mab/9649?site-search-type=Products&N=4294956287&Ntt=cetyl-histone+h3+%28lys9%29&fromPage=plp

Tri-Methyl-Histone H3 (Lys27)
Validation Refs. from the manufacturer's website: https://www.cellsignal.com/products/primary-antibodies/tri-methyl-histone-h3-lys27-c36b11-rabbit-mab/9733

Histone H3
Validation Refs. from the manufacturer's website: https://www.cellsignal.com/products/primary-antibodies/histone-h3-3h1-rabbit-mab/9717

HA-Tag
Validation Refs. from the manufacturer's website: https://www.biolegend.com/en-us/search-results/purified-anti-ha-11-epitope-tag-antibody-11374

Annexin V-APC
Validation Refs. from the manufacturer's website: https://www.biolegend.com/en-us/explore-new-products/apc-annexin-v-8144

GAPDH
Validation Refs. from the manufacturer's website: https://www.abcam.com/gapdh-antibody-6c5-loading-control-ab8245.html

H3K27ac
Validation Refs. from the manufacturer's website: https://www.abcam.com/histone-h3-di-methyl-k79-antibody-chip-grade-ab3594.html

Flag-Tag
Validation Refs. from the manufacturer's website: https://www.sigmaaldrich.com/US/en/product/sigma/f1804

Tubulin
Validation Refs. from the manufacturer's website: https://www.sigmaaldrich.com/US/en/product/sigma/t9026?context=product

RNF5, validated by knockdown and knockout in this study
Validation Refs. from the manufacturer's website: https://www.scbt.com/p/rnf5-antibody-22b3

HSP90
Validation Refs. from the manufacturer's website: https://www.scbt.com/p/hsp-90alpha-beta-antibody-f-8

Caspase 3
Validation Refs. from the manufacturer's website: https://www.scbt.com/p/caspase-3-antibody-31a1067?requestFrom=search

Calregulin
Validation Refs. from the manufacturer's website: https://www.scbt.com/p/calregulin-antibody-a-9?requestFrom=search

RBBP4, validated by knockdown in this study
Validation Refs. from the manufacturer's website: https://www.novusbio.com/products/rbbp4-rbap48-antibody-13d10_nbp1-41201

HRP-conjugated anti-Mouse
Validation Refs. from the manufacturer's website: https://www.jacksonimmuno.com/catalog/products/115-035-062

HRP-conjugated anti-Rabbit
Validation Refs. from the manufacturer's website: https://www.jacksonimmuno.com/catalog/products/111-035-045/Goat-Rabbit-IgG-HL-Horseradish-Peroxidase

Eukaryotic cell lines

Policy information about cell lines

Cell line source(s)	Human HEK293T and A375 cells were obtained from ATCC (CRL-11268, CRL-1619). U937 (ATCC, CRL-1593.2) and K562 (ATCC, CCL-243) cells were kindly provided by Prof. Yuval Shaked (Technion, Israel), Kasumi-1 (ATCC, CRL-2724) cells were kindly provided by Prof. Tsila Zuckerman (Rambam hospital, Israel), MV4-11 (ATCC, CRL-9591), GRANTA (DSMZ—German Collection of Microorganisms and Cell Cultures, ACC 342), THP-1 (ATCC, TIB-202), and MEC-1 (DSMZ—German Collection of Microorganisms and Cell Cultures, ACC 497) cells were kindly provided by Dr. Netanel Horowitz (Rambam hospital, Israel), MOLM-13 (DSMZ—German Collection of Microorganisms and Cell Cultures, ACC 554) cells were kindly provided by Dr. Ani Deshpande (SBP Discovery Institute, USA), KG-1a (ATCC, CCL-246.1), HL-60 (ATCC, CCL-240), Jurkat (ATCC, TIB-152), RPMI 8226 (ATCC CCL-155) and HAP-1 (Horizon Discovery, C631) cells were a kind gift from Prof. Ciechanover (Technion, Israel).
Authentication	Cell lines were obtained from verified sources and authenticated in our Genomic Shared Resources at SBP using short tandem repeat (STR) analysis. Allele profiles for each line were matched those maintained in the Expasy Cellosaurus STR database (https://web.expasy.org/cellosaurus/) using CLASTR v1.4.4
Mycoplasma contamination	All the cell lines tested negative for mycoplasma contamination (MycoAlert-Lonza).
Commonly misidentified lines (See ICLAC register)	No commonly misidentified cell lines were used.

Animals and other organisms

Policy information about studies involving animals; ARRIVE guidelines recommended for reporting animal research

Laboratory animals	All mice used were either C57BL/6 or NOD/SCID (NOD.CB17-Prkdcscid/J) genetic background and 6–10-weeks-old female mice. Animals were housed in 5 mice per cage and maintained under controlled temperature (22.5°C) and illumination (12 h dark/light cycle) conditions. All experimental animal procedures were approved by the Institutional Animal Care and Use Committee of Sanford Burnham Prebys Medical Discovery Institute (approval AUF 16-028).
Wild animals	No wild animals were used in this study.
Field-collected samples	This study did not involve samples collected from the field.
Ethics oversight	All experimental animal procedures were approved by the Institutional Animal Care and Use Committee of Sanford Burnham Prebys Medical Discovery Institute (approval AUF 16-028).

Note that full information on the approval of the study protocol must also be provided in the manuscript.

Human research participants

Policy information about studies involving human research participants

Population characteristics	AML patient samples were obtained from Scripps MD Anderson, La Jolla, CA (IRB-approved protocol 13-6180) and written informed consent was obtained from each participant, and Rambam Health Campus Center, Haifa, Israel (IRB-approved protocol 0372-17). Fresh blood samples were obtained by peripheral blood draw, PICC line, or central catheter. Filgrastim-mobilized peripheral blood cells were collected from healthy donors and cryopreserved with DMSO. PBMCs were isolated by centrifugation through Ficoll-PaqueTM PLUS (17-1440-02, GE Healthcare). Residual red blood cells were removed using RBC Lysis Buffer for Human (Alfa Aesar, cat. # J62990) according to the manufacturer's instructions. The final PBMC pellets were resuspended in Bambanker serum-free freezing medium (Wako Pure Chemical Industries, Ltd.) and stored under liquid N2.<br>MLL-AF9 patient-derived xenograft (PDX) samples were obtained from the Jeremias Lab, Munich, Germany and were cultured in IMDM medium with 20% BIT (Stem cell Technologies), human cytokines and StemRegenin 1 (SR1) and UM171.
Recruitment	These patients were not recruited for this study.
Ethics oversight	AML patient samples were obtained from Scripps MD Anderson, La Jolla, CA (IRB-approved protocol 13-6180) and from Rambam Health Campus Center, Haifa, Israel (IRB-approved protocol 0372-17).

Note that full information on the approval of the study protocol must also be provided in the manuscript.

Flow Cytometry

Plots

Confirm that:

- [x] The axis labels state the marker and fluorochrome used (e.g. CD4-FITC).
- [x] The axis scales are clearly visible. Include numbers along axes only for bottom left plot of group (a 'group' is an analysis of identical markers).
- [x] All plots are contour plots with outliers or pseudocolor plots.
- [x] A numerical value for number of cells or percentage (with statistics) is provided.

Methodology

Sample preparation	For annexin V and propidium iodide (PI) staining: cells were collected in FACS tubes, washed twice with ice-cold 1×phosphate-buffered saline (PBS), and resuspended in 100 µL PBS. Annexin V-APC (1.4 µg/mL) was added for 15 minutes at room temperature in the dark. Then, cells were washed in PBS and resuspended in 200 µL PBS and PI (50 µg/mL) was added. Samples were then analyzed by flow cytometry (BD LSRFortessa™, BD Biosciences).<br>For cell cycle analysis, distribution of cells in each phase of the cell cycle was analyzed by propidium iodide staining (Merck). Briefly, one million cells were washed twice with cold PBS and fixed in 70% ethanol in PBS at 4°C overnight. Cells were washed, pelleted by centrifugation, and treated with RNase A (100 µg/mL) and propidium iodide (40 µg/mL) at room temperature for 30 min<br>For flow cytometric quantification of MLL-AF9 GFP+ cells: Retro-orbital bleedings were performed 15 and 28 days post tail vein injections of GFP-MLL-AF9–transformed WT or Rnf5–/– cells. Red blood cells were lysed using Red Blood Cell lysing buffer (Sigma-Aldrich), then washed with PBS and resuspended in bone marrow wash buffer (2% FBS in PBS). Sytox blue (Invitrogen) was added to exclude dead cells. Cells were then analyzed by flow cytometry (BD LSRFortessa™, BD Biosciences).
Instrument	All data were collected on an LSRFortessa (BD Biosciences).
Software	All data were collected on FACSDive Software (BD) and analyzed using FlowJo v10.1 and v9.9 Software (Tree Star).
Cell population abundance	For cell sorting, highest purity method was used in BD FACSDiva (8.0.2) Flow Cytometer and over 98% post sorting purity was verified through flow cytometry analysis. For population abundance in FACS analysis experiments, see Supplementary information (Source data).
Gating strategy	MLL-AF9 GFP+ analysis: GFP, Sytox blue<br>Cell cycle analysis: PI<br>Annexin V and propidium iodide staining: Annexin V-APC, PI

- [x] Tick this box to confirm that a figure exemplifying the gating strategy is provided in the Supplementary Information.
