# Supplementary material for: The ubiquitin ligase RNF5 determines acute myeloid leukemia growth and susceptibility to histone deacetylase inhibitors
Source: Nat Commun. 2021 Sep 13;12:5397. doi: 10.1038/s41467-021-25664-7 (PMC8437979; doi:10.1038/s41467-021-25664-7)

Unmodified blots Fig. 1

Fig. 1b

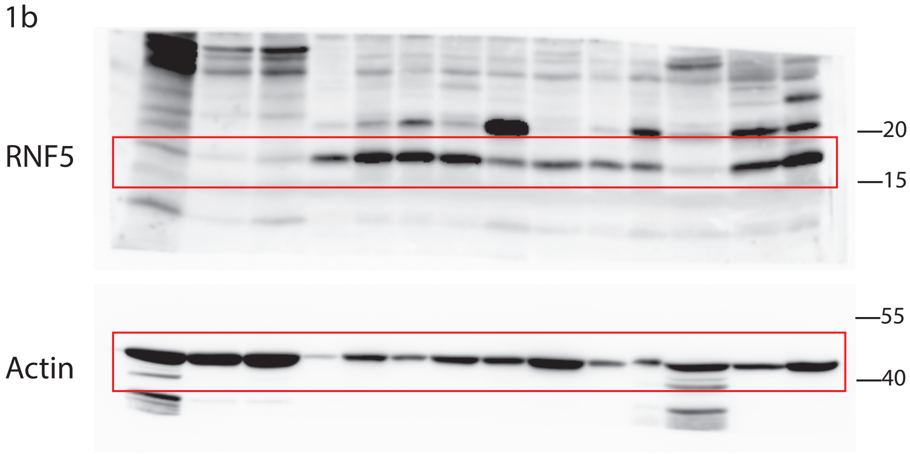

Fig. 1f

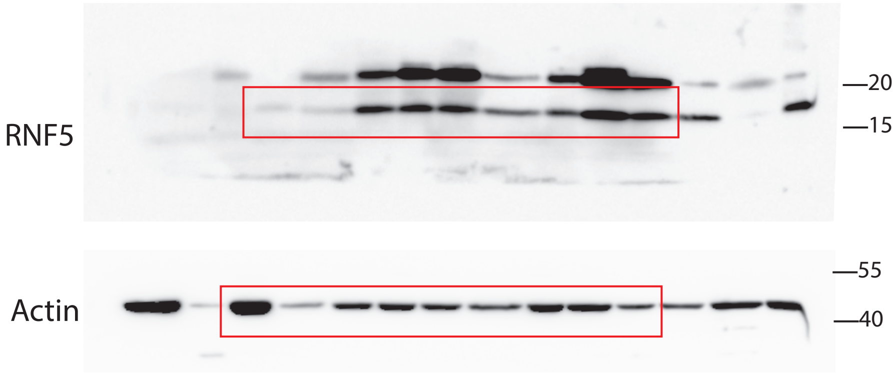

Fig. 1h

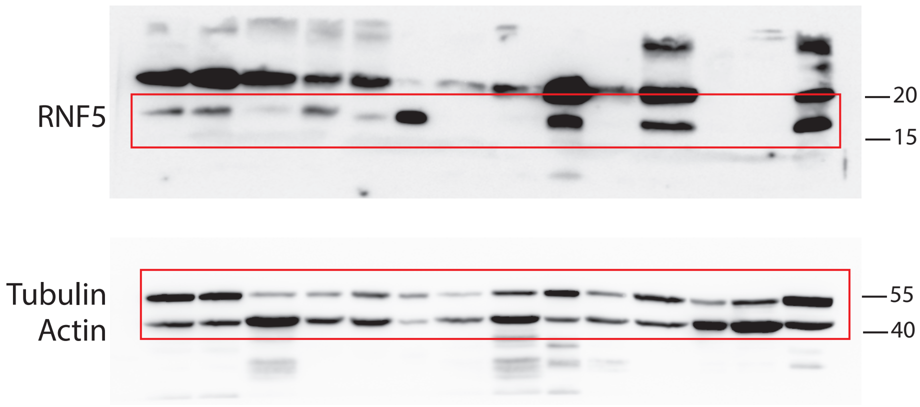

Unmodified blots Fig. 2

Fig. 2c

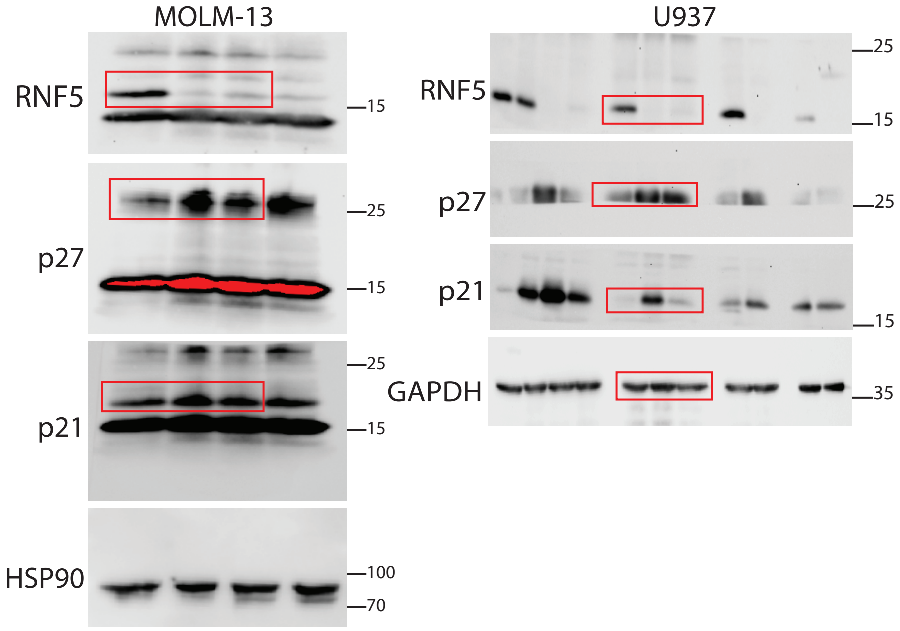

Fig. 2g

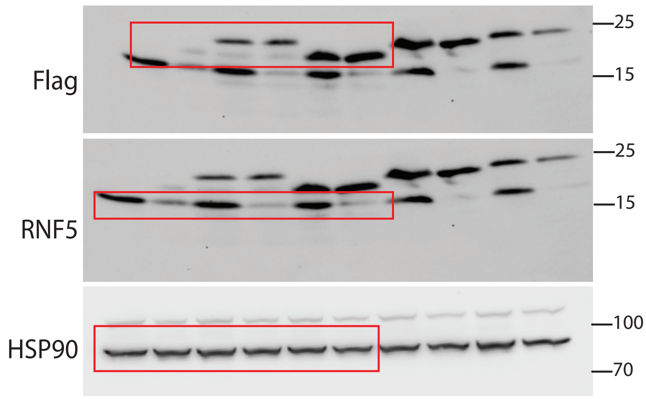

Fig. 2e

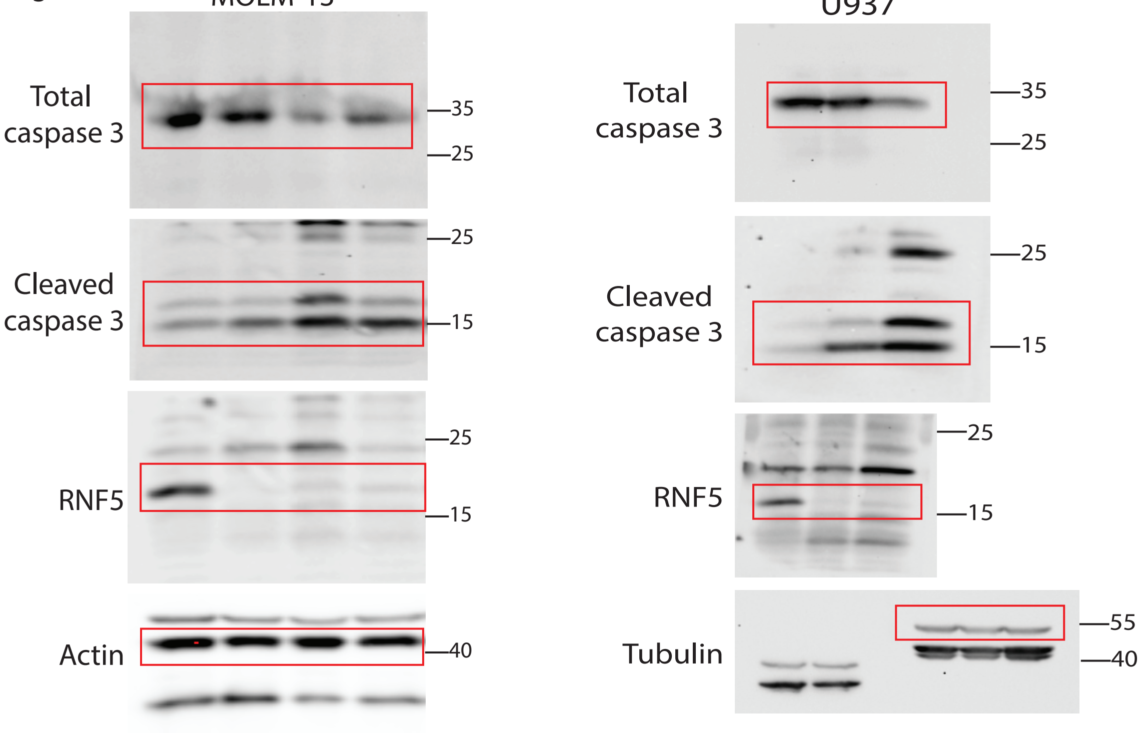

Fig. 2i

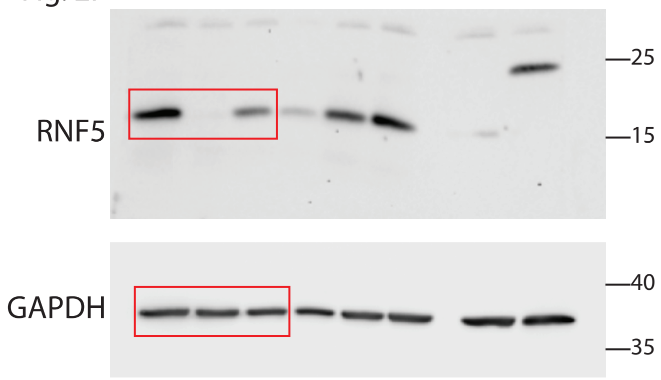

Fig. 2l

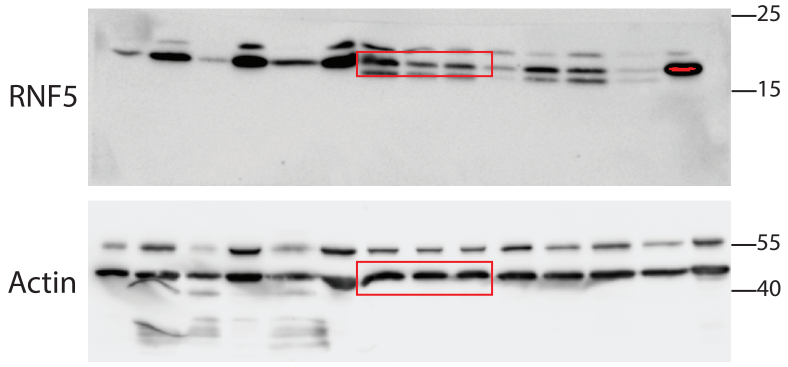

Unmodified blots Fig. 3

Fig. 3a

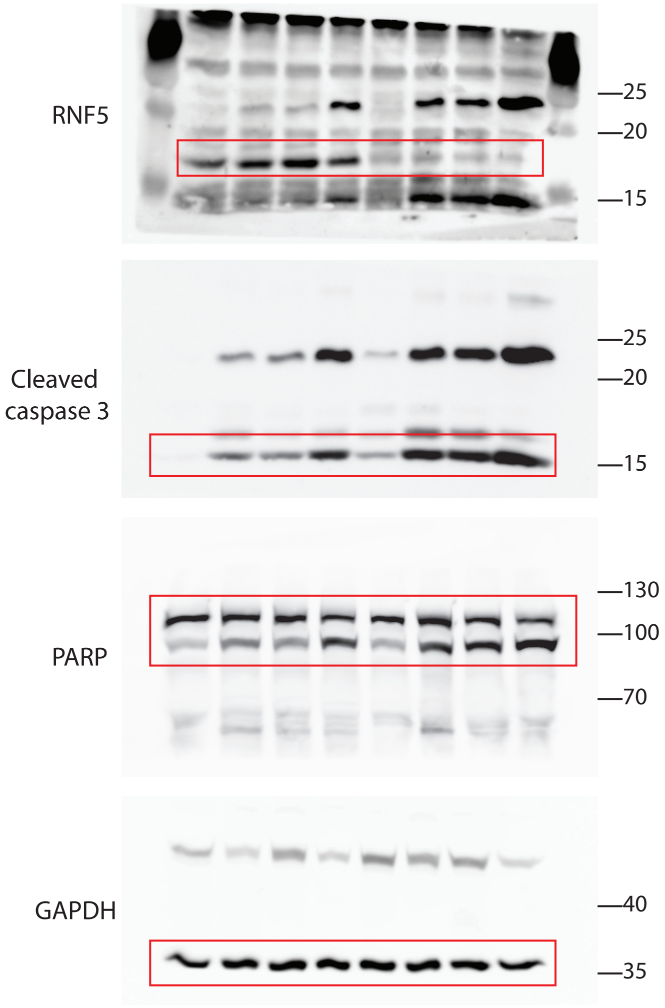

Fig. 3b

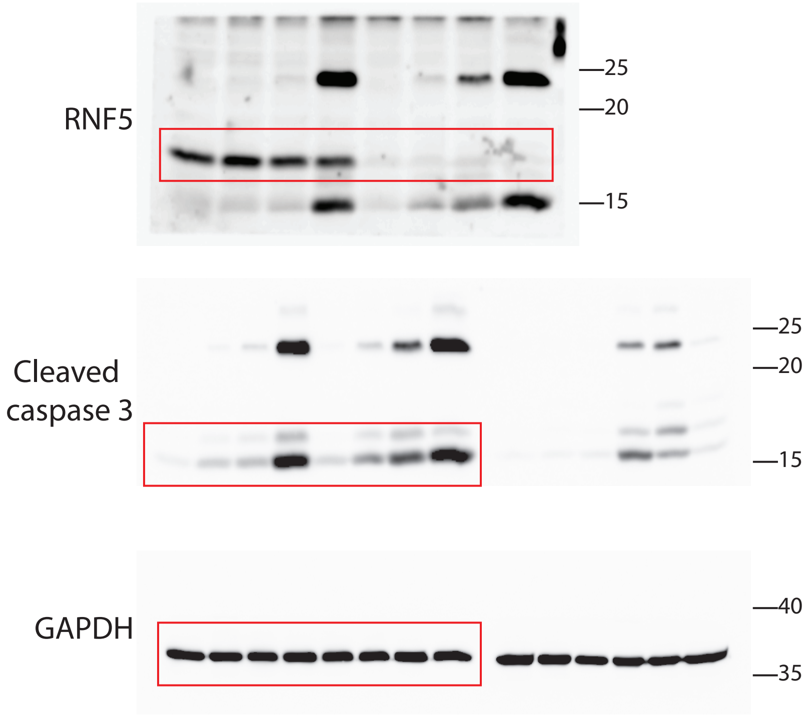

Fig. 3e

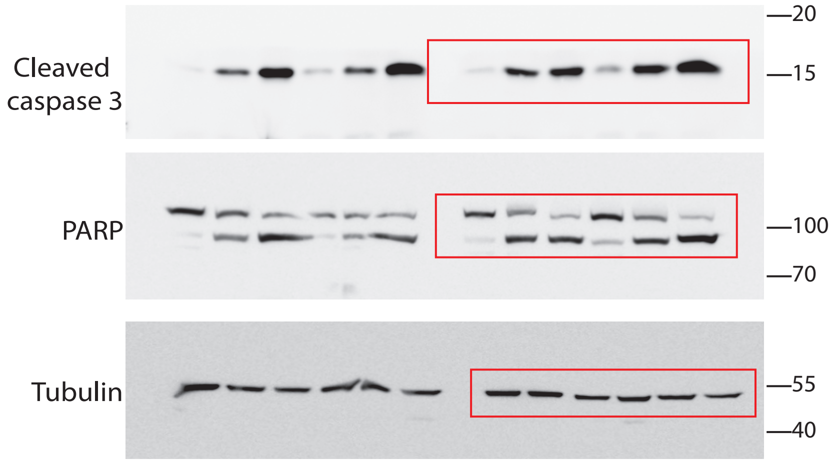

Unmodified blots Fig. 5

Fig. 5h

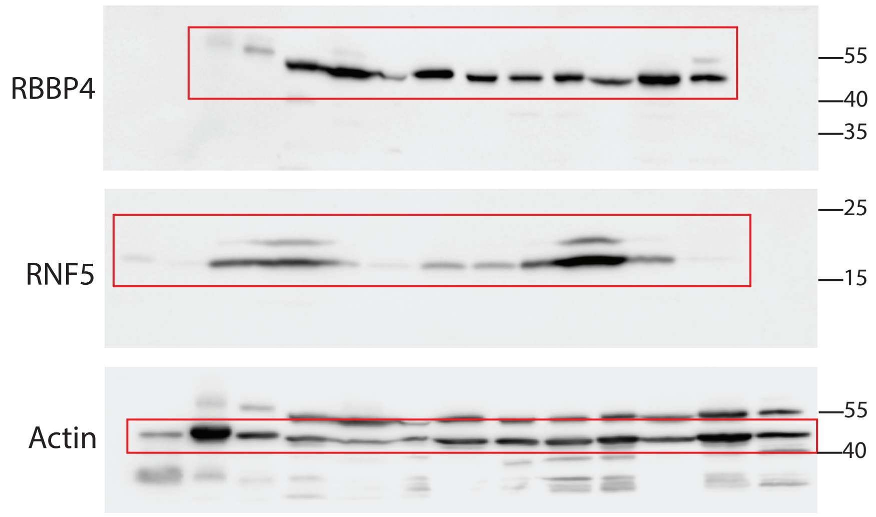

Fig. 5k

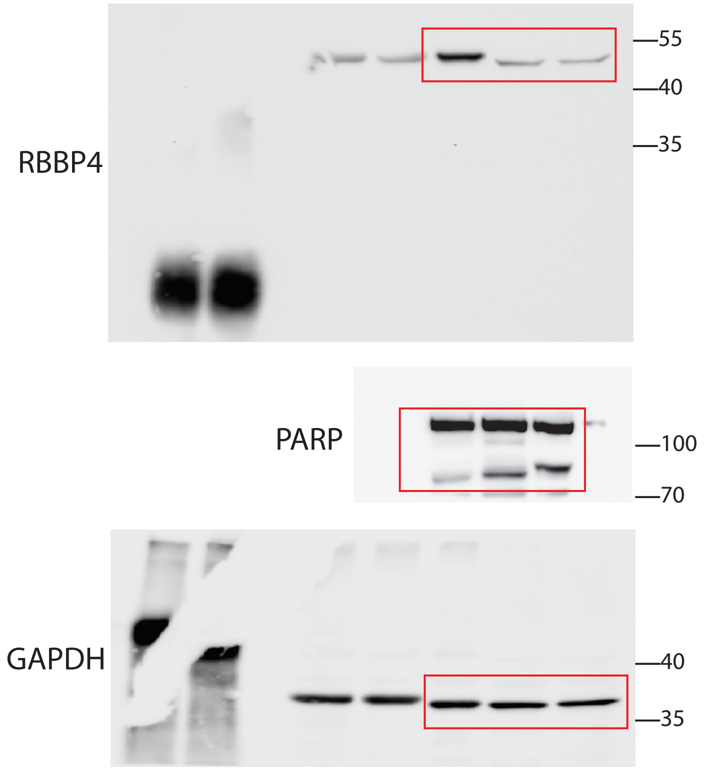

Unmodified blots Fig. 6

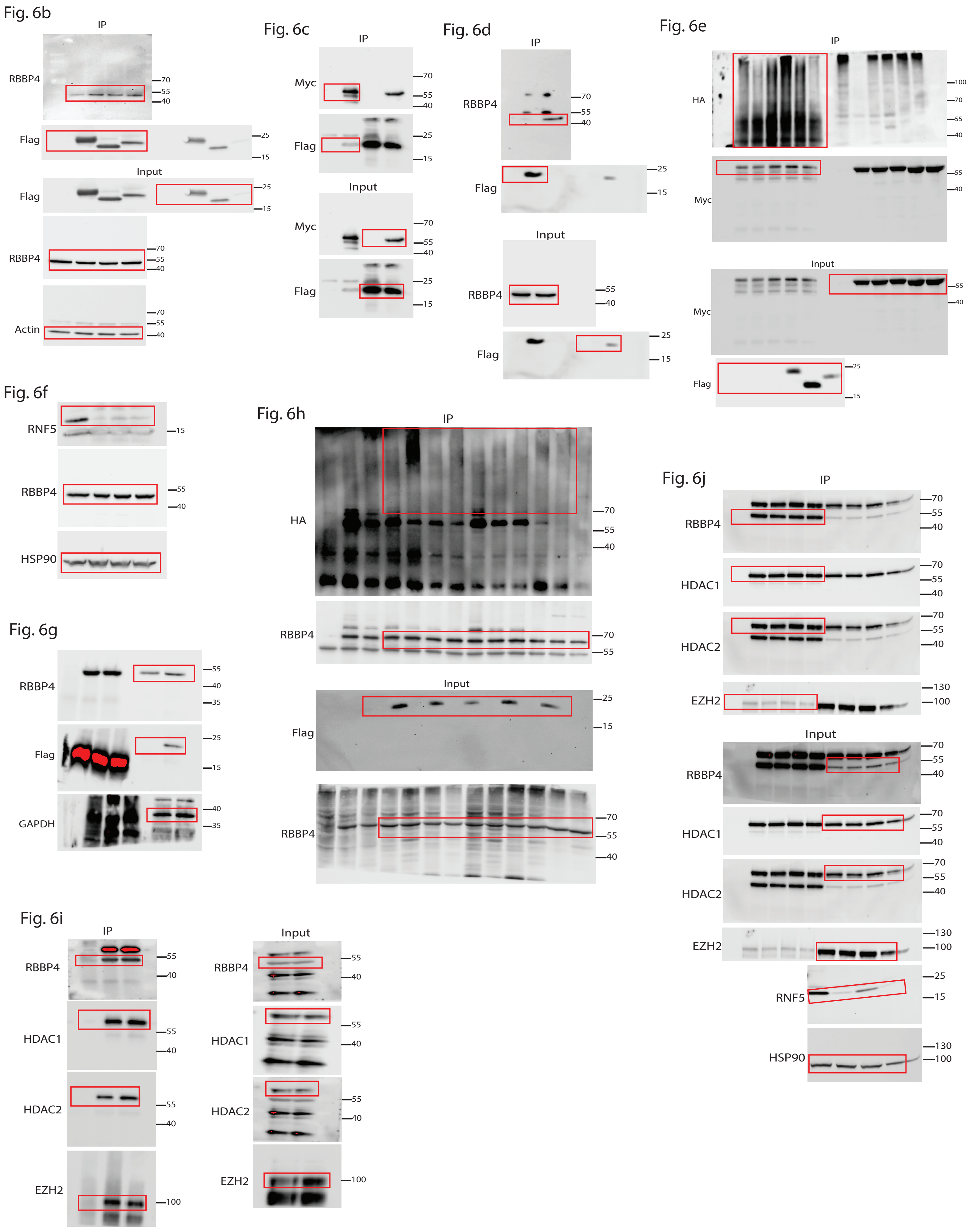

Unmodified blots Fig. 7

Fig. 7f

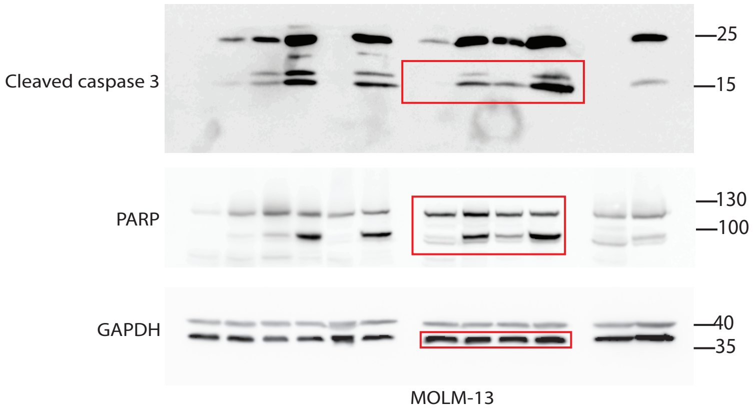

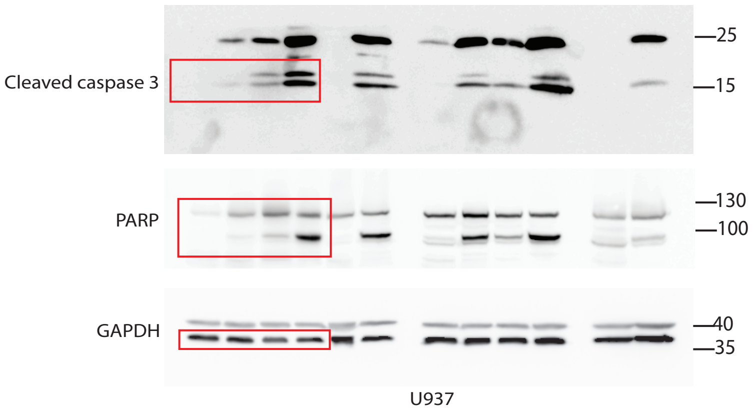

Fig. 7l

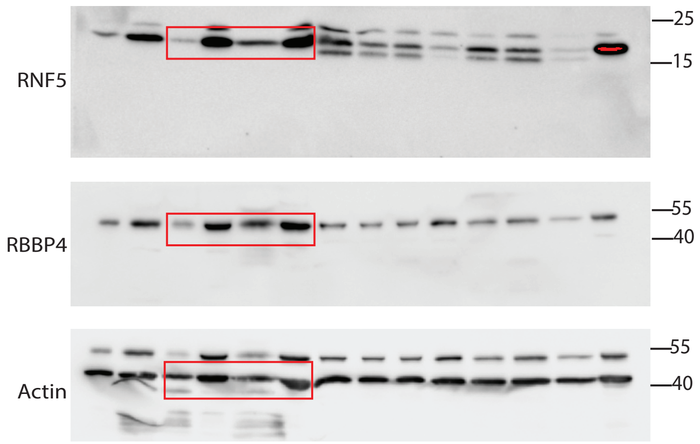

Unmodified blots Supplmentary Fig. 1

Fig. S1b

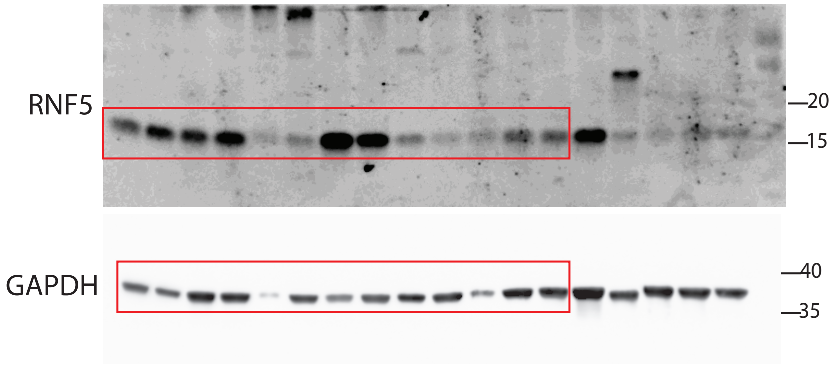

Fig. S1c

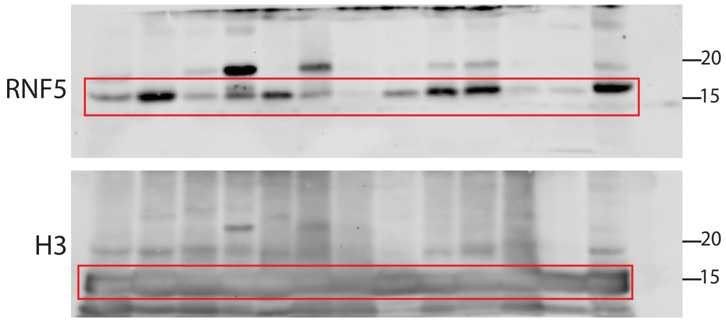

Fig. S1d

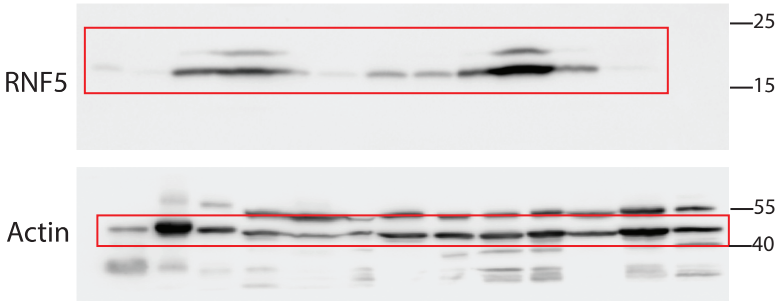

Fig. S1j

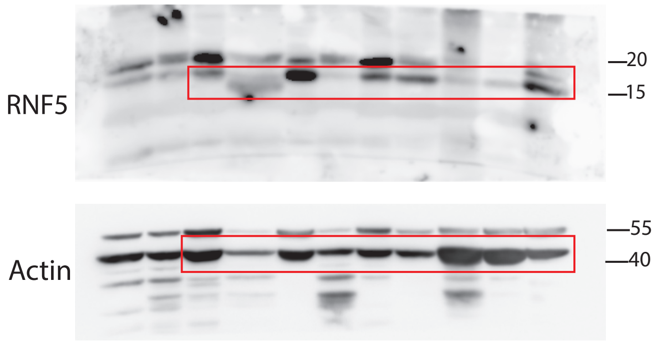

Unmodified blots Supplmentary Fig. 2

Fig. S2b

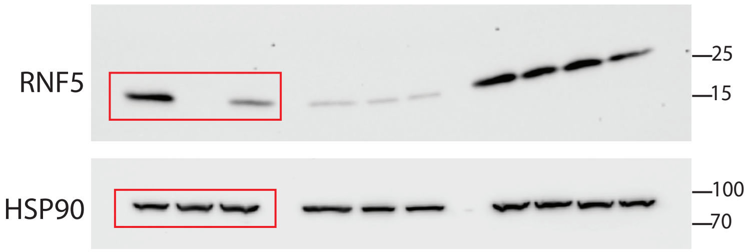

Fig. S2c

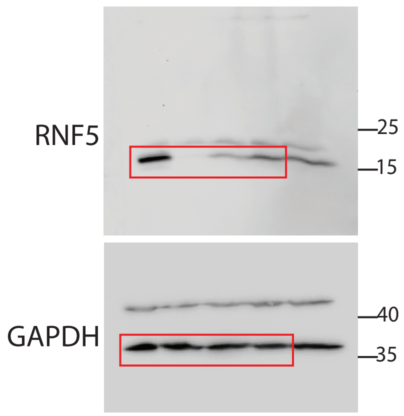

Fig. S2d

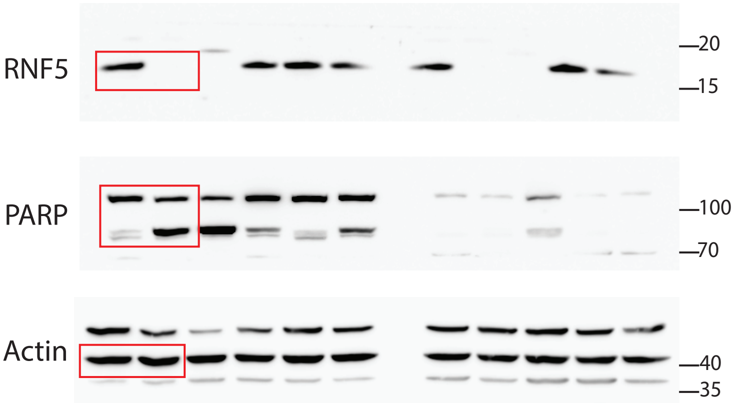

MOLM-13

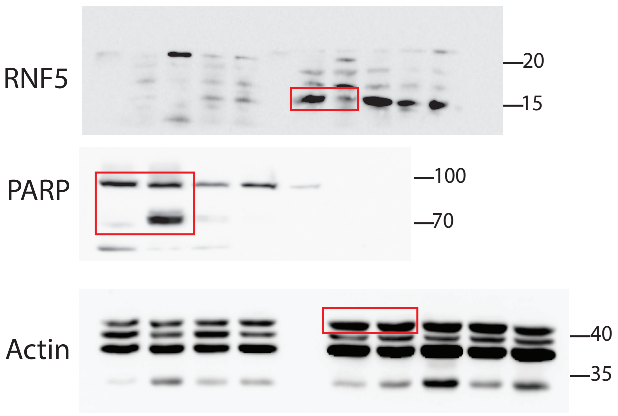

U937

Fig. S2h

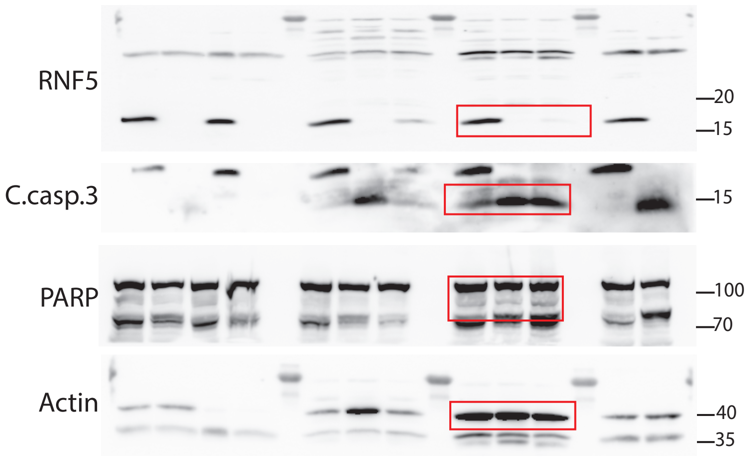

HL-60

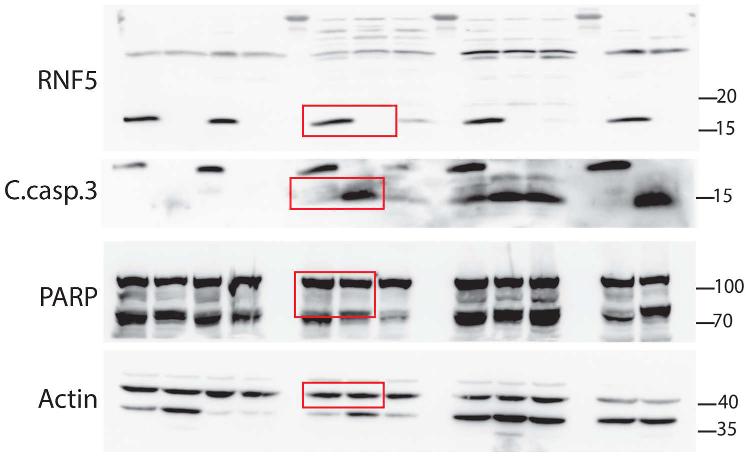

THP-1

Unmodified blots Supplmentary Fig. 4

Fig. S4a

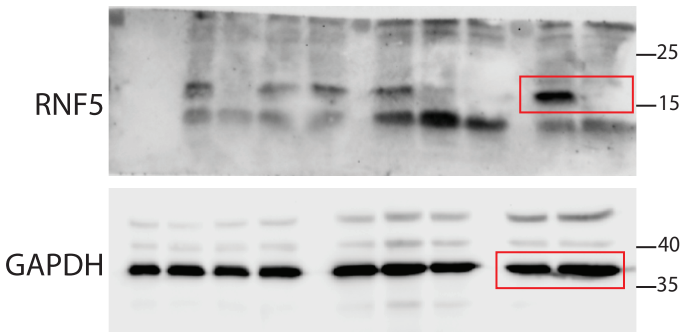

Fig. S4d

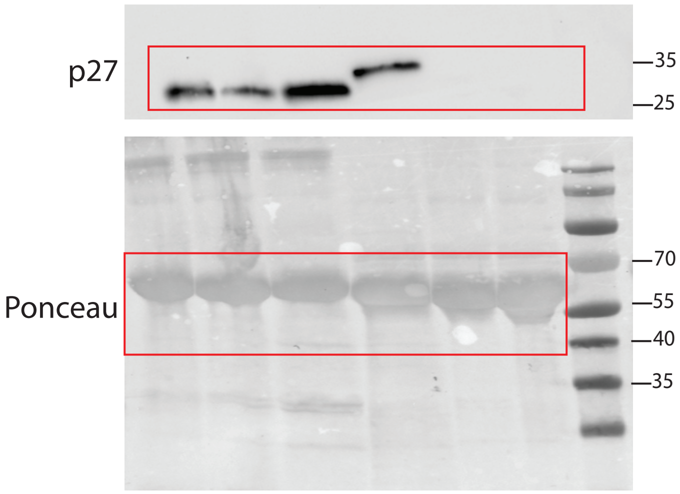

Fig. S4e

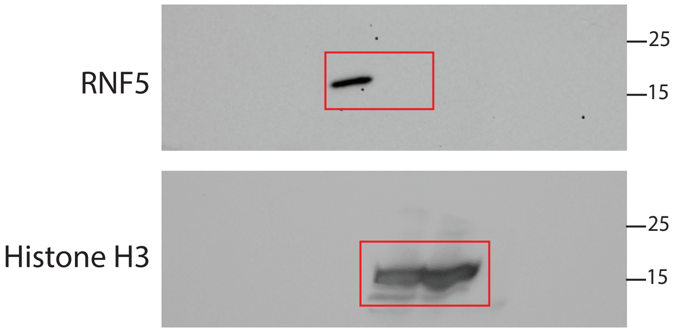

Unmodified blots Supplmentary Fig. 5

Fig. S5k

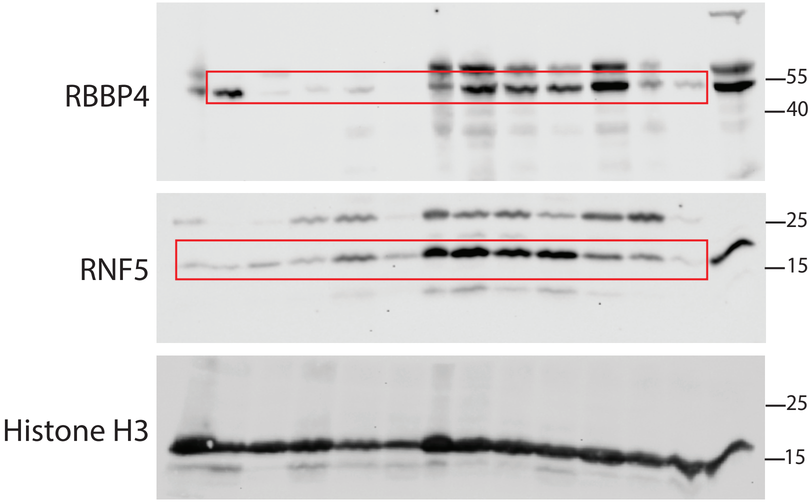

Fig. S5m

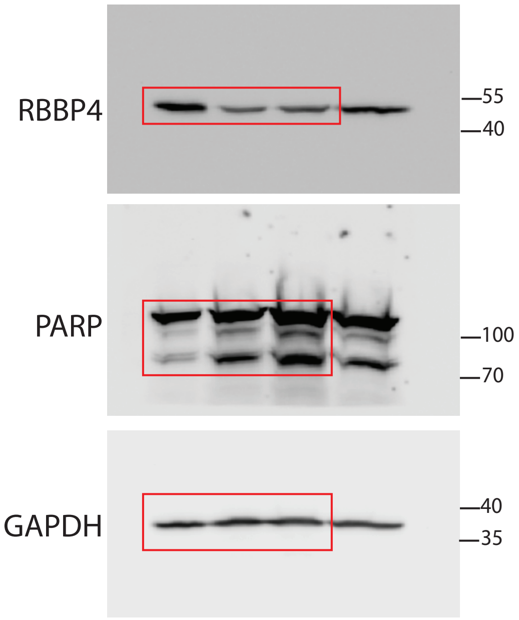

Fig. S5n

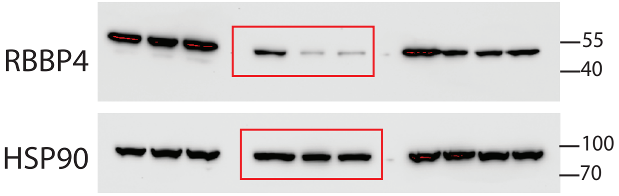

Fig. S5p

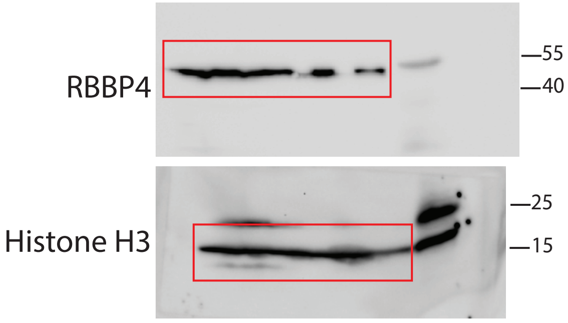

Unmodified blots Supplmentary Fig. 6

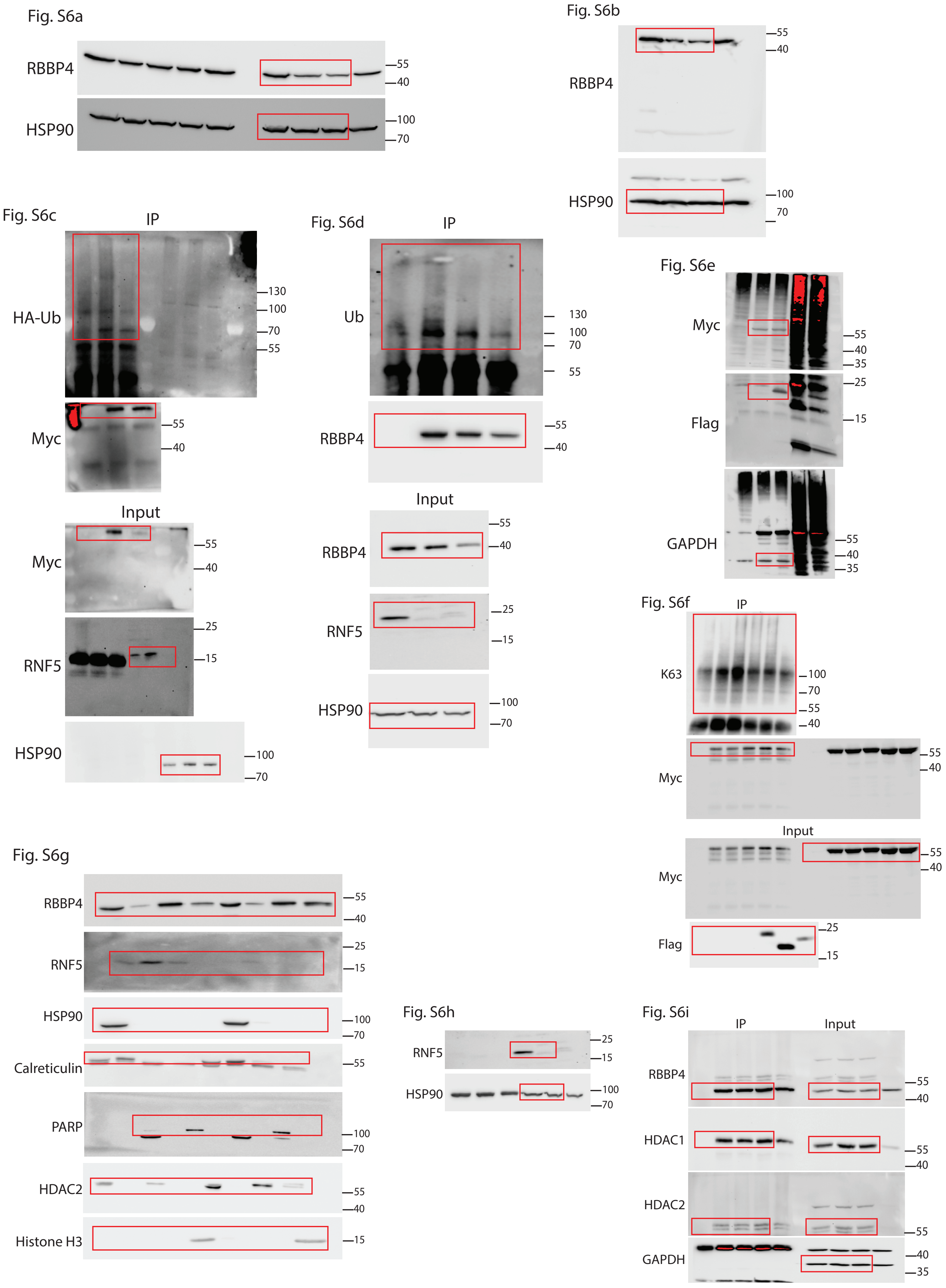

Unmodified blots Supplmentary Fig. 7

Fig. S7a, c

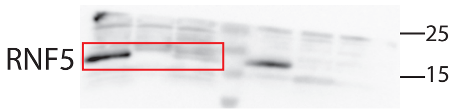

Fig. S7d

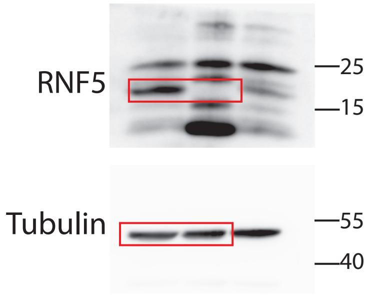

Fig. S7e

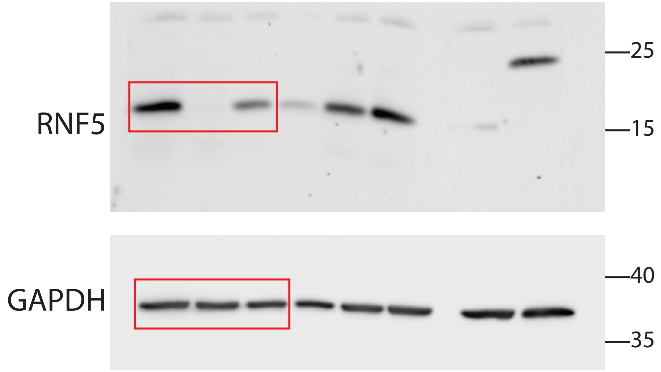

Fig. S7j

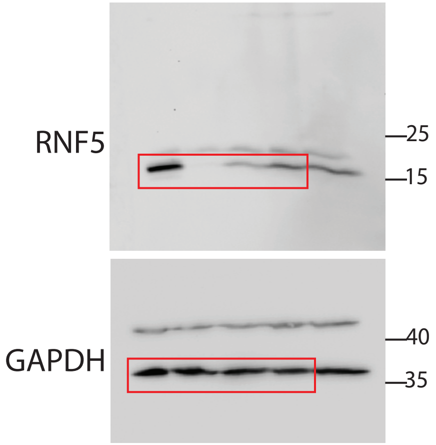

Fig. S7k

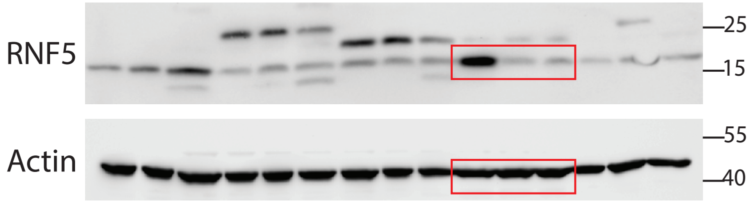

Supplement: Supplementary file 8 — Source Data [file 41467_2021_25664_MOESM8_ESM.zip › Western blot Gels.pdf]
